# Mutational characterization and mapping of the 70S ribosome active site

**DOI:** 10.1093/nar/gkaa001

**Published:** 2020-02-03

**Authors:** Anne E d’Aquino, Tasfia Azim, Nikolay A Aleksashin, Adam J Hockenberry, Antje Krüger, Michael C Jewett

**Affiliations:** 1 Interdisciplinary Biological Sciences Program, Northwestern University, Evanston, IL 60208, USA; 2 Department of Chemical and Biological Engineering, Northwestern University, Evanston, IL 60208, USA; 3 Chemistry of Life Processes Institute, Northwestern University, Evanston, IL 60208, USA; 4 Center for Synthetic Biology, Northwestern University, Evanston, IL 60208, USA; 5 Center for Biomolecular Sciences, University of Illinois at Chicago, Chicago, IL 60607, USA; 6 Robert H. Lurie Comprehensive Cancer Center, Northwestern University, Chicago, IL 60611, USA; 7 Simpson Querrey Institute, Northwestern University, Chicago, IL 60611, USA

## Abstract

The synthetic capability of the *Escherichia coli* ribosome has attracted efforts to repurpose it for novel functions, such as the synthesis of polymers containing non-natural building blocks. However, efforts to repurpose ribosomes are limited by the lack of complete peptidyl transferase center (PTC) active site mutational analyses to inform design. To address this limitation, we leverage an *in vitro* ribosome synthesis platform to build and test every possible single nucleotide mutation within the PTC-ring, A-loop and P-loop, 180 total point mutations. These mutant ribosomes were characterized by assessing bulk protein synthesis kinetics, readthrough, assembly, and structure mapping. Despite the highly-conserved nature of the PTC, we found that >85% of the PTC nucleotides possess mutational flexibility. Our work represents a comprehensive single-point mutant characterization and mapping of the 70S ribosome's active site. We anticipate that it will facilitate structure-function relationships within the ribosome and make possible new synthetic biology applications.

## INTRODUCTION

The *Escherichia coli* ribosome is the molecular machine that polymerizes α-amino acids into polypeptides using information encoded in messenger RNAs (mRNAs). This machine is composed of two distinct subunits: the large (50S) subunit, responsible for accommodating tRNA-amino acid monomers, catalyzing peptide bond formation and excreting polypeptides, and the small (30S) subunit, primarily responsible for decoding the mRNA. The active site of the ribosome, or the peptidyl transferase center (PTC), residing in the 23S ribosomal RNA (rRNA) of the 50S subunit, is composed primarily of conserved catalytic rRNA nucleotides, but has been demonstrated to possess ribosomal protein as well (1–4).

**Figure 1.**
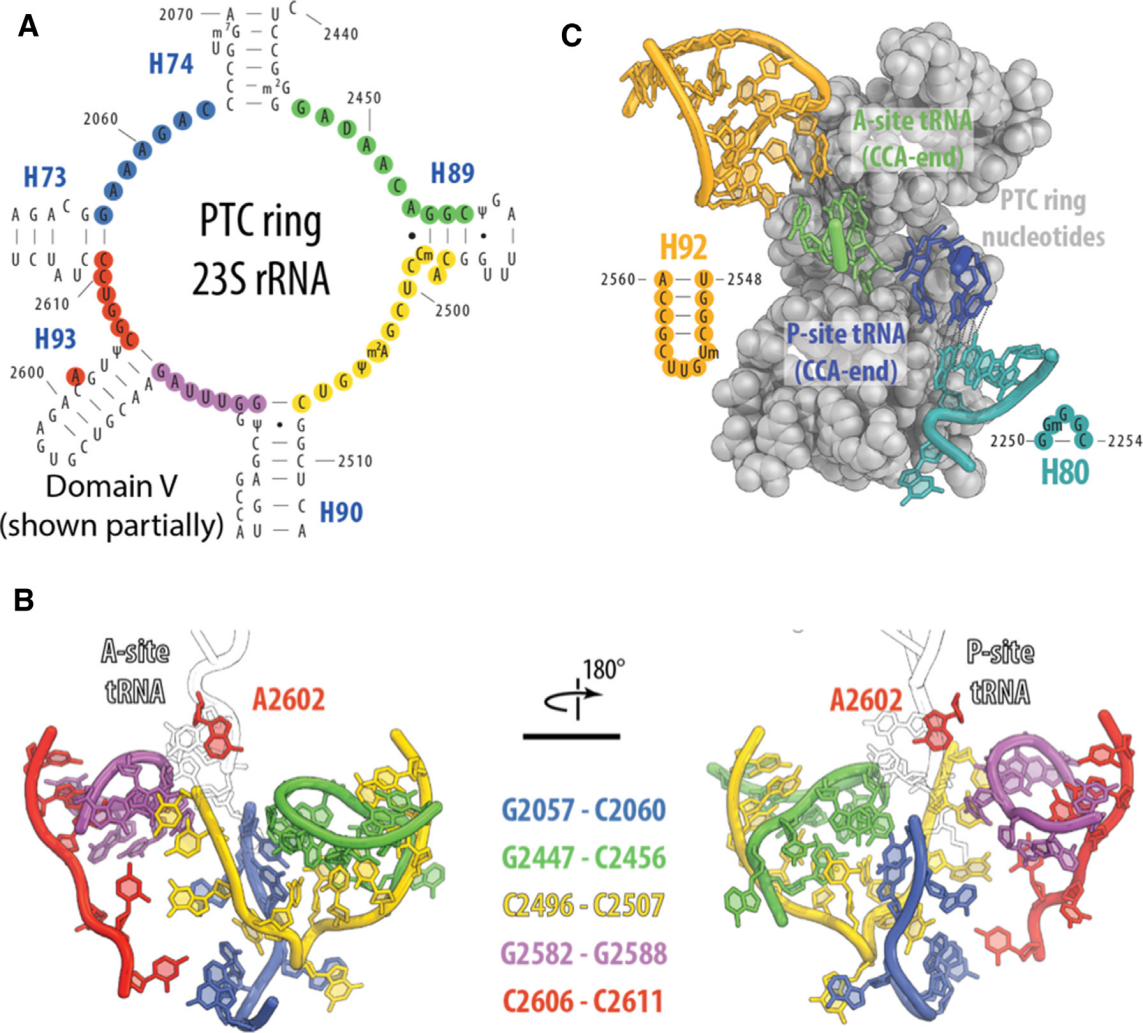
The ribosome's peptidyl transferase center (PTC) is important for translation and can be studied *in vitro*. (**A**) Secondary structure and (**B**) crystal structure model of the PTC-ring nucleotides probed in this study. (**C**) Secondary structure and crystal structure model of the A- and P-loop nucleotides probed in this study. Ribosome structure accessed from PDB ID: 4YBB, with tRNAs from PDB ID: 1VY4.

Previous works have revealed that many key catalytic functions of the ribosome are executed by its RNA components in the PTC; making the ribosome an ancient ribozyme (5). For example, the rRNA nucleotides of the PTC play a key role in positioning the CCA ends of the aminoacyl (A)-site and peptidyl (P)-site tRNA monomers to catalyze peptide bond formation and facilitate peptide release (6). Additional studies suggest that ribosomal proteins may contribute to catalytic function as well (1–4). Most notably, a number of L27 residues are positioned to interact with the peptidyl-tRNA, potentially stabilizing the 3′ ends of the tRNA substrates in the PTC for catalysis (1). Within the PTC, sets of key rRNA nucleotides are arranged as rings and loops, with the central PTC-ring, A-loop and P-loop playing pivotal roles in translation (5,7,8) (Figure 1). The central PTC-ring (defined in our study as G2057–C2063, G2447–C2456, C2496–C2507, G2582–G2588, A2602 and C2606–C2611) surrounds the A- and P-site tRNA monomers and has been implicated in antibiotic binding (9), tRNA positioning (10) and peptide stalling (11,12). As their names suggest, the A-loop (defined in our study as U2548–A2560) is essential in interacting with A-site tRNA during translation, while the P-loop (defined in our study as G2250–C2254) makes contacts with P-site tRNA (7,13–15). The A- and P-loops are co-located on either side of the central PTC-ring, above the peptide exit tunnel (Figure 1). All of these nucleotides have previously been identified as essential catalytic bases, as their identities are highly conserved (16).

Pioneering *in vivo* and *in vitro* studies of the *E. coli* ribosome's active site have provided a foundational understanding of ribosome structure, function, and mechanism (17–22). However, we lack a comprehensive understanding of the PTC in its entirety, in part, because a complete functional mutational analysis does not exist. This gap in knowledge is rooted in several challenges. One challenge, for example, includes insufficient high-throughput methods to synthesize and characterize a large number of ribosomal mutations. As a result, existing ribosomal mutation studies typically focus only on a few mutations at a time (i.e. one to six in depth characterizations per paper) (23,24), use characterization techniques that can be difficult to compare (spanning *in vitro* biochemistry, *in vivo* genetics, computational modelling, antibiotic resistance probing and more), and sometimes examine different bacterial species. This has led to a segmented and heterogeneous image of the ribosome's mutational space (Supplementary Table S1). Another challenge is the highly-conserved nature and functional importance of many active site nucleotides. Characterization of mutations at these locations has proven difficult as nucleotide changes confer deleterious phenotypes (25–27). Thus, despite insights gained from crystal structures and decades of biochemical analyses, we still currently lack direct mutational and functional knowledge for many key nucleotides in the ribosome's active site. A comprehensive mutational map of the rRNA within the ribosome's active site would facilitate antibiotic resistance studies (25), enable active site and rRNA engineering efforts (28–30), and build on our current understanding of structure–function relationships within the ribosome (28).

To circumvent cell-viability constraints (31), a cell-free (32–38), or *in vitro*, ribosome synthesis approach could be used for identifying structurally and functionally critical sites in the ribosome useful for both basic biology and future ribosome engineering advances (39). For example, the elegant ‘atomic mutagenesis’ approach developed by Polacek and colleagues has helped unravel the detailed contributions of rRNA nucleotides in protein synthesis (40–42). In previous work, we developed and optimized a different approach for use with *E. coli* ribosomes; the integrated synthesis, assembly and translation (iSAT) platform for the *in vitro* construction and characterization of ribosomes (43–46). The iSAT platform leverages a ribosome-free S150 crude extract to enable the efficient transcription of template-derived rRNA. Importantly, iSAT co-activates the processes of rRNA synthesis and processing, ribosome assembly, and translation in a one-pot reaction, mimicking natural *in vivo* processes. The iSAT system therefore provides a unique and powerful approach for the interrogation and manipulation of *E. coli* ribosomes in a cell-like environment. This system contrasts with previous approaches for *in vitro* ribosome reconstitution, which have played important roles in elucidating our understanding of the ribosome (24), but are unable to incorporate synthetic *in vitro* transcribed 23S rRNA of the large subunit into highly active *E. coli* ribosomes (39,41,47–50). Key advantages of the iSAT platform include no wild-type ribosome contamination, facile and rapid mutant construction and testing, and a reaction environment that closely resembles the cell.

In this work, we use the iSAT platform to rapidly characterize ribosomal active site mutations. Importantly, these catalytic regions of the ribosome are highly conserved, implying that these interactions are essential for peptide-bond formation. Thus, studies of these regions will help further define the structural and functional importance of these particular sequences in the ribosome, and which sequences are permissible to change. Specifically, we probed all nucleotides in the catalytically critical PTC-ring, A-loop, and P-loop by: (i) constructing single point mutations at every possible rRNA position within these loops (180 total mutations); (ii) testing their translational activity *in vitro*; (iii) assaying translation readthrough impairments using premature stop codon as a proxy for translation accuracy or release factor fidelity (decreased binding affinity or hydrolysis) (23,51–53) and; (iv) characterizing ribosomal assembly. Finally, we analyzed our mutational activity data in the context of the three-dimensional ribosome structure by mapping our findings directly onto the crystal structure (Figure 1). We report the first, to our knowledge, comprehensive molecular dissection of the ribosome's active site in the context of mutational flexibility, and the development of a high-throughput and standardized workflow for rapidly constructing and characterizing rRNA mutants. We envision these findings to be a stepping stone for both basic scientists and synthetic biologists to target, study, and engineer single or multiple ribosomal nucleotides.

## MATERIALS AND METHODS

### Plasmid construction

The 7300-bp plasmid pT7rrnB carries an *Escherichia coli* rRNA operon, *rrnB*, under the control of the T7 promoter and the ampicillin resistance gene as a selective marker. All ribosomal mutant plasmids are derivatives of pT7rrnB carrying single point mutations in the 23S rRNA gene. Briefly, site-directed mutagenesis was used to construct each individual point mutant. Nucleotide point mutations were introduced into primers and amplified using pT7rrnB as a template for PCR amplification. PCR products were blunt end ligated, transformed into DH5α using electroporation, and plated onto LB-agar/ampicillin plates at 37°C. Plasmid was recovered from resulting clones and sequence confirmed.

Similarly, premature stop codon readthrough constructs were generated by introducing a premature stop sequence (UAG, UGA, of UAA) into primers, and amplified using pJL1-sfGFP as a template for PCR amplification. PCR products were blunt end ligated, transformed into DH5α using electroporation, and plated onto LB-agar/Kanamycin plates at 37°C. Readthrough controls were generated from reporter constructs by introducing all possible stop codon permutations (UGA, UAA and UAG) at various positions within the reporter (Figure 3A). All constructs were verified by DNA sequencing.

### Sequence alignment and analysis

A dataset consisting of 1,614 pre-aligned and phylogenetically arranged 23S sequences were downloaded from the All Species Living Tree Project (version 123, compiled using the SILVA reference database LSUref123) (54). This dataset included the *E. coli* sequence (AJ278710) that was used as a reference to find regions of interest in the full species alignment using custom scripts (available at https://github.com/adamhockenberry/23s-alignment-LTP). All species were used in visualizations, but entropy calculations included only analysis of ungapped sequences. Specifically, for each position in the alignment of a region of interest we first removed any sequence where that position was denoted by a ‘-’ character. With the remaining sequences, we calculated the entropy values (*H*) as:

$$\begin{equation*}H = - \mathop \sum \nolimits_{i\ \in ({A,U,G,C})} {p_i}{\rm{log}}\left( {{p_i}} \right)\end{equation*}$$

where the probability of nucleotide *i* (*p_i_*) comes from the counts of nucleotide *i* divided by the number of all non-gapped sequences at that position. In this formulation, *H* has a minimum of 0 when all sequences in an alignment column are one nucleotide and a maximum of ∼1.386 when all nucleotides are equally likely (i.e. occurring with a probability of 0.25).

### Strain culture and harvest

*Escherichia coli* cells for S150 extract and TP70 preparation were grown in 10L of 2xYPTG in a fermenter (Sartorius) (Supplementary Figure S11). MRE600 strain was grown at 37°C. Cells were harvested at OD_600_ = 2.8–3.0, washed twice in S150 lysis buffer (20 mM Tris–chloride pH 7.2 at 4°C, 100 mM ammonium chloride, 10 mM magnesium chloride, 0.5 mM EDTA, 2 mM DTT), pelleted, and flash frozen at −80°C using liquid nitrogen for storage. Buffer was added at a ratio of 5 ml of buffer per 1 g of cells. 200 μl of Halt Protease Inhibitor Cocktail (Thermo Fisher Scientific Inc.) and 75 μl RNase Inhibitor (Qiagen) were added for every 4 g of cells in the suspension. The cells were lysed at ∼20 000 psi with an EmulsiFlex-C3 homogenizer (Avestin). An equivalent dose of RNase Inhibitor and 3 μl of 1 M DTT per milliliter were added to the lysate prior to two clarification spins at 30 000 g and 4°C for 30 min. Supernatant equivalent to S30 crude extract was recovered and gently layered into Ti45 ultracentrifuge tubes on top of an equivalent volume of sucrose cushion, buffer B (20 mM Tris–HCl (pH 7.2 at 4°C), 100 mM NH_4_Cl, 10 mM MgCl_2_, 0.5 mM EDTA, 2 mM DTT, 37.7% sucrose). Samples were then centrifuged (at 35 000 rpm in Ti70 rotor) and 4°C for 20 h. Supernatant was recovered for S150 extract, and the remaining clear ribosome pellet was gently washed and resuspended in buffer C (10 mM Tris–OAc (pH 7.5 at 4°C), 60 mM NH_4_Cl, 7.5 mM Mg(OAc)_2_, 0.5 mM EDTA, 2 mM DTT). Concentration of resuspended ribosomes was determined from *A*_260_ NanoDrop readings (1 *A*_260_ unit of 70S = 24 pmol 70S (55)). Ribosomes were then aliquoted and flash-frozen for use as purified 70S ribosomes and for purification of native rRNA and r-proteins.

### Component preparation

S150 crude cell-free extracts, *E. coli* 70S ribosomes, total protein of 70S ribosomes (TP70) and T7 RNA polymerase (RNAP) were prepared as previously reported (44,56). S150 and TP70 were prepared from MRE600 cells. Protein concentrations of each S150 extract were measured using Bradford assay with bovine serum albumin (BSA) as a standard.

### iSAT reactions

iSAT reactions of 15 μl were set-up as previously described (44). Briefly, reactions were prepared in polymerase chain reaction tubes with optically clear flat caps and incubated at 37°C in a CFX96 real-time thermal cycler (Bio-Rad). iSAT reactions contained reporter protein plasmids encoding superfolder GFP (sfGFP). Green fluorescence of sfGFP was monitored using the CFX96real-time thermal cycler as (excitation: 450–490 nm, emission: 510–530 nm). Additives were included at the described final concentrations. Specifically, crowding agent (2% PEG-6000) and reducing agent (2 mM DTT) were added to each reaction. iSAT reactions for S150 extracts were optimized for concentrations of magnesium glutamate to maximize reaction productivity and minimize consumption of parts (Supplementary Figure S12). sfGFP quantification was performed as previously reported (43), using measurements of relative fluorescence units (RFU) from CFX96 real-time thermal cycler (BioRad, Hercules, CA, USA) and BioTek Synergy 2 plate reader (Winooski, VT, USA). RFU values were converted to molar concentration using a linear standard curve made in-house by expressing ^14^C-leucine labeled sfGFP in *E. coli* PANOx CFPS reactions and relating RFUs to trichloracetic acid precipitable soluble protein yield.

### Ribosome sedimentation analysis

Sucrose gradients were prepared from gradient buffer (20 mM Tris–HCl (pH 7.5 at 4°C), 100 mM NH_4_Cl, 10 mM MgCl_2_) with 10 and 40% sucrose in SW41 polycarbonate tubes using a Biocomp Gradient Master. Gradients were placed in SW41 buckets and chilled to 4°C overnight. Meanwhile, approximately 3 × 15 μl iSAT reactions were prepared and incubated at 37°C, for 2 h. Reactions were flash frozen in liquid nitrogen. The next day, reactions were pooled and 45 μl of iSAT reactions were diluted to 100 μl with gradient buffer, and carefully loaded onto chilled gradients. The gradients were ultra-centrifuged to 39 000 rpm for 2.5 h at 4°C, using an Optima L-80 XP ultracentrifuge (Beckman-Coulter) at maximum acceleration and braking. Gradients were analyzed with a Piston Gradient Fractionator (Biocomp). Traces of A254 readings versus elution volumes were obtained for each gradient, with readings adjusted to match baselines based on blank sucrose readings. iSAT reactions without the operon plasmid were performed to establish a background reading that was subtracted from experimental traces. Gradient fractions were collected and analyzed for rRNA content by gel electrophoresis in 1% agarose and imaged in a GelDoc Imager (Bio-Rad) (Supplementary Figure S13). Ribosome profile peaks were identified based on the rRNA content as representing 30S or 50S subunits, 70S ribosomes, or polysomes, as well as a control peak with purified 70S. To calculate the area under each curve, Riemann sums were taken with the 30S x-axis boundaries ranging from ∼13 mm gradient distance to ∼21 mm, the 50S *x*-axis boundary ranging from ∼22 to ∼28 mm, the 70S *x*-axis boundary ranging from ∼30 to 40 mm, and the polysomes *x*-axis boundary ranging from ∼42 to 59 mm. Sums between each X-axis coordinate were taken, and totals were calculated for the given boundaries.

### iSAT ribosome purification

Several (∼8) 15 μl iSAT reactions were prepared and incubated for 2 h at 37°C, then pooled together. Purified 70S *E. coli* ribosomes were recovered as previously described (44), with pelleted iSAT ribosomes resuspended in iSAT buffer, aliquoted and flash-frozen.

### Nucleotide distance calculations

Nucleotide distances were measured between the average center of each nucleotide to the average center of A76 of each respective tRNA and the attached amino acid residue of each the A-site and P-site tRNA molecules. Distances were calculated from the structure file of PDB ID: 4YBB, with tRNAs from PDB ID: 1VY4 (57) (Supplementary Table S5 and S6).

## RESULTS

### Examining mutational flexibility of PTC rRNA *in vitro*

**Figure 2.**
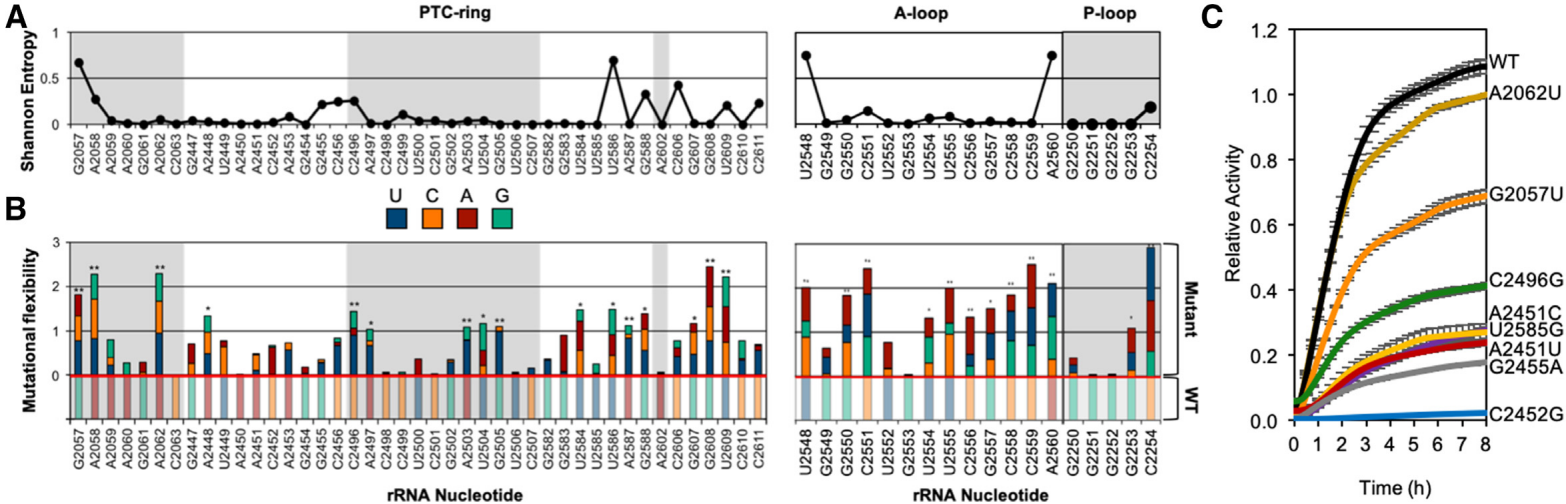
The ribosome's peptidyl transferase center (PTC) is amenable to mutation, despite high sequence conservation. (**A**) Shannon entropy plot representing the conservation of PTC nucleotides across 1614 of bacterial and archael species. All large subunit (LSU) sequences were taken from alignments found at the Silva database (43). Shannon entropy values of zero represent 100% conservation across all species. Despite the high conservation of the ribosome's PTC, there is high plasticity within its catalytic core. (**B**) Mutational flexibility of each PTC mutation relative to the activity of WT iSAT ribosomes. Nucleotides are color coded according to the legend. The original WT nucleotide activity is normalized to 1, and resides below the red line on the graph. Each possible nucleotide change at the corresponding position is color coded in the bars above the red line, with bar size representing relative activity. Single asterisks are placed above nucleotides wherein the sum of mutant activity (mutational flexibility) results in activity ≥1 (PTC-ring: G2057, A2058, A2062, A2448, C2496, A2497, A2503, U2504, G2505, U2584, U2586, A2587, G2588, G2607, G2608 and U2609; A- and P-loops: U2548, G2550, C2551, U2554, U2555, C2556, G2557, C2558, C2559, A2560, G2253, C2254). A second asterisk is placed above nucleotides wherein at least one nucleotide mutation results in activity ≥ 75% of WT activity (PTC-ring: G2057, A2058, A2062, C2496, A2503, G2505, A2587, G2608 and U2609; A- and P-loops: U2548, G2550, C2551, U2555, C2556, C2558, C2559, A2560, C2554). (**C**) Protein synthesis curves for representative nucleotide mutations have been included in this graph: wild-type, high activity (A2062U and G2057U), medium activity (C2496G and A2451C), medium-low activity (U2585G and A2451U), and low activity (G2455A and C2452G) mutants were chosen. Translation rates for representative PTC mutants in this study are represented in Supplementary Table S2. For simplicity and ease of visualization, only a subset of 180 nucleotide mutation kinetic curves are included on the graph and the X-axis is restricted to 8 h.

The goal of this study was to use the iSAT platform to construct and characterize ribosomal active site mutants and generate a functional map of mutational flexibility. However, the ribosome's active site has evolved to accurately and efficiently process α-amino acid monomers using catalytic rRNA, that we would expect to exhibit high levels of conservation and would be less permissible, or flexible, to mutation. In fact, previous work has demonstrated *in vivo* that many nucleotide changes to highly-conserved nucleotides are detrimental (25), but the ribosome can still withstand some changes at select positions (58) (Supplementary Table S1). As a first step in characterizing the ribosome's active site, we quantitatively evaluated conservation at every nucleotide position within the PTC. Large subunit (LSU) sequences were taken from the Silva rRNA database and aligned at PTC-nucleotide positions (54). Sequences were aligned for 1614 species of bacteria and archaea (Supplementary Figure S1 and S2) and Shannon Entropy values were calculated (Figure 2A). Shannon Entropy scores are akin to variance scores (though we caution that they ignore phylogenetic relatedness), with a Shannon Entropy of zero representing zero variance (100% conservation across the 1614 species). Any values above zero indicate that evolutionary changes have occurred and result in multiple nucleotides within a given site in the alignment. As expected, the entire PTC active site (PTC-ring, A-loop, and P-loop) exhibited a high-level of conservation, with ∼75% of the nucleotide positions possessing a Shannon Entropy value at or near zero. Although there is high conservation, all rRNA positions are not equally conserved.

While the PTC active site exhibits high levels of nucleotide conservation, we can assess mutational flexibility at each rRNA nucleotide position by building rRNA mutants in the iSAT system. We constructed iSAT reactions, as previously described (43–46), possessing wild-type and all 180 mutant ribosomes, separately, and measured reporter protein biosynthesis yields via fluorescent activity over the course of 16–20 h (Figure 2B, C and Supplementary Figure S3). When looking at all 180 mutants, we observe a wide range of translation rates *in vitro*. Importantly, these rates are representative of overall activity, and have been summarized in numerical order in tabular form in Supplementary Table S2 (also Figure 2C, Table 1, Supplementary Figure S3 and S4). Relative activity was subsequently calculated to compare performance of each mutant by normalizing wild-type protein synthesis yields to one and mutant yields to the normalized wild-type yields. An overall mutational flexibility score was then determined for each nucleotide position by adding the relative activities of every possible point mutation. The highest mutational flexibility score of three indicates that all three nucleotide changes possess wild-type activity, while the lowest mutational flexibility score of zero indicates that all three nucleotide changes preclude any protein synthesis (Supplementary Table S3).

**Table 1.** Bulk translation rates of wild-type and representative mutant 70S iSAT ribosomes. Bulk translation rates for iSAT ribosomes were determined from protein synthesis kinetics curves, for reactions after 2 h incubations

rRNA mutation	Bulk translation rate (μM protein/h)
WT	1.03 ± 0.03
A2062U	1.02 ± 0.02
G2608C	0.57 ± 0.03
G2057U	0.63 ± 0.02
U2449C	0.52 ± 0.02
C2452A	0.52 ± 0.02
C2496G	0.31± 0.02
A2451C	0.15 ± 0.01
U2585G	0.20 ± 0.01
A2451U	0.14 ± 0.01
G2455A	0.13 ± 0.01
C2452G	0.004 ± 0.01

Despite the highly-conserved nature of the ribosome's active site, the majority (>85%) of the PTC-ring nucleotides possessed some degree of flexibility to mutational changes (one or more mutations at that position permitted full-length protein synthesis, determined by protein activity), as did 80% of A- and P-loop nucleotides (Figure 2). Of the 43 PTC-ring nucleotides, 16 positions (G2057, A2058, A2062, A2448, C2496, A2497, A2503, U2504, G2505, U2584, U2586, A2587, G2588, G2607, G2608 and U2609) possessed a mutational flexibility score ≥ 1. And across the A- and P-loop nucleotides, 12 positions (U2548, G2550, C2551, U2554, U2555, C2556, G2557, C2558, C2559, A2560, G2253, C2254) resulted in a mutational flexibility score ≥ 1. Additionally, 9 PTC-ring nucleotides (G2057, A2058, A2062, C2496, A2503, G2505, A2587, G2608 and U2609) and 9 A- and P-loop nucleotides (U2548, G2550, C2551, U2555, C2556, C2558, C2559, A2560, C2554) possessed at least one nucleotide mutation that resulted in ≥75% of WT activity.

We then tested the degree to which our findings relate to natural sequence diversity of 23S rRNA sequences by correlating mutational flexibility for individual sites with their Shannon Entropy values measured across the 1614 species. For the PTC-ring, we found a significant (*P* = 0.025) but weak (*R*^2^ = 0.117) relationship, indicating that sequence diversity explains only a minor fraction of the observed variation in mutational flexibility (Supplementary Figure S5). For the A- and P-loops, we found a non-significant (*P* = 0.086) and weak (*R*^2^ = 0.173) relationship, indicating that the sequence diversity does not explain the observed variation in mutational flexibility. In total, these results illustrate a large degree of mutational flexibility that exists within the PTC and the difficulty in predicting mutational flexibility solely from nucleotide conservation.

### Characterizing PTC mutant ribosome translation readthrough

**Figure 3.**
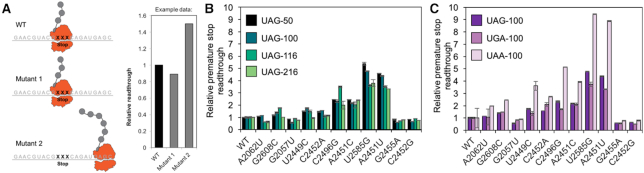
Ribosomal PTC mutations increase stop-codon readthrough. (**A**) Schematic of translation fidelity assay using premature stop codon constructs. Assays were adapted from O’Connor *et al.* (37). Premature stop codon readthrough from wild-type ribosomes was normalized to 1. Mutant ribosome premature stop codon readthrough was quantified through fluorescence and set relative to WT. Mutants with lower fidelity (higher readthrough of premature stop codons) produce higher relative sfGFP titers. (**B**) UAG stop codon readthrough at amino acid position 50, 100, 116 and 216. (**C**) UAG, UGA and UAA stop codon readthrough at amino acid position 100. Relative activity in translation fidelity assays using premature stop codons was assessed using sfGFP fluorescence. The pJL1-sfGFP plasmid possessing a UAG stop codon at the specified locations were introduced into iSAT reactions as the reporter plasmid along with the mutant or wild-type rRNA plasmids. Values represent averages and error bars represent one standard deviation from the mean, with *n*≥ 3 for n number of independent reactions.

With the PTC exhibiting a high degree of mutational flexibility, we wondered if mutants of highly-conserved nucleotides that possessed observable translational activity were exhibiting readthrough impairments during protein synthesis. Readthrough impairments could be a product of either decreased translation accuracy, or release factor fidelity (decreased binding affinity or hydrolysis) (59–61). Previously, mutations in the active site of the *E. coli* ribosome were reported to have a negative impact on translation readthrough and fidelity (23,24,53,62), suggesting that our mutant ribosomes might have the same issues. To assess whether our rRNA mutants’ functionality was being impacted by impaired translation readthrough, we carried out a series of experiments involving premature stop codon readthrough adapted from previously-reported assays (23) (Figure 3A). Specifically, the readthrough assay measures fluorescence output of iSAT reactions using sfGFP reporter constructs separately possessing UAG premature stop codons at amino acid positions 50, 100, 116 or 216 of sfGFP, and comparing three different stop codons (UAG, UGA and UAA) all positioned at amino acid 100 of sfGFP. Readthrough efficiencies were determined by comparing relative active sfGFP produced from iSAT reactions using each rRNA mutant construct to wild-type ribosome constructs for each reporter. These relative readthrough efficiencies were then normalized by each mutants’ translation activity. We tested whether ribosomes with mutations possessing high, medium, and low activity (PTC-ring: A2062U, G2608C, G2057U, U2559C, C2452A, C2496G, A2451C, U2585G, A2451U, G2455A, C2452G; A-loop: C2559A, C2551A, U2552G; and P-loop: C2254G, G2253C, G2251A) could readthrough engineered stop codons in sfGFP mRNA (Figure 3; Supplementary Figures S6 and S7).

Our results demonstrate that PTC-ring mutations C2496G, A2451C, U2585G and A2451U exhibit a high-degree of stop codon readthrough (Figure 3B and C), while the A- and P-loop mutations we probed maintain minimal readthrough, similar to wild-type ribosomes (Supplementary Figures S6 and S7). Importantly, the overall readthrough trends across the mutants are mirrored across each unique premature stop codon construct (the same four ribosome mutants exhibiting a higher-degree of readthrough compared to wild-type ribosomes); however, normalized relative readthrough signals for UAA were greater than those for stop codons UAG and UGA. We hypothesize that these results are attributed to both release factors, RF1 and RF2, recognizing the UAA stop codon during wild-type translation (while UAG is only recognized by RF1 and UGA is only recognized by RF2) (63,64). This increased recognition may result in higher UAA termination efficiencies in our system and thus reduced fluorescence signals for wild-type ribosomes reading this construct. Thus, when relative readthrough is normalized across the mutants compared to wild-type, any small amount of readthrough from a mutant ribosome is further amplified with this stop codon. In summary, the results of our readthrough assay emphasize the important role that PTC-ring nucleotides play in translation readthrough, whether it be accuracy or termination fidelity, as compared to A- and P-loop bases.

### Incorporation of ribosomes with PTC active site mutations into functional polysomes

**Figure 4.**
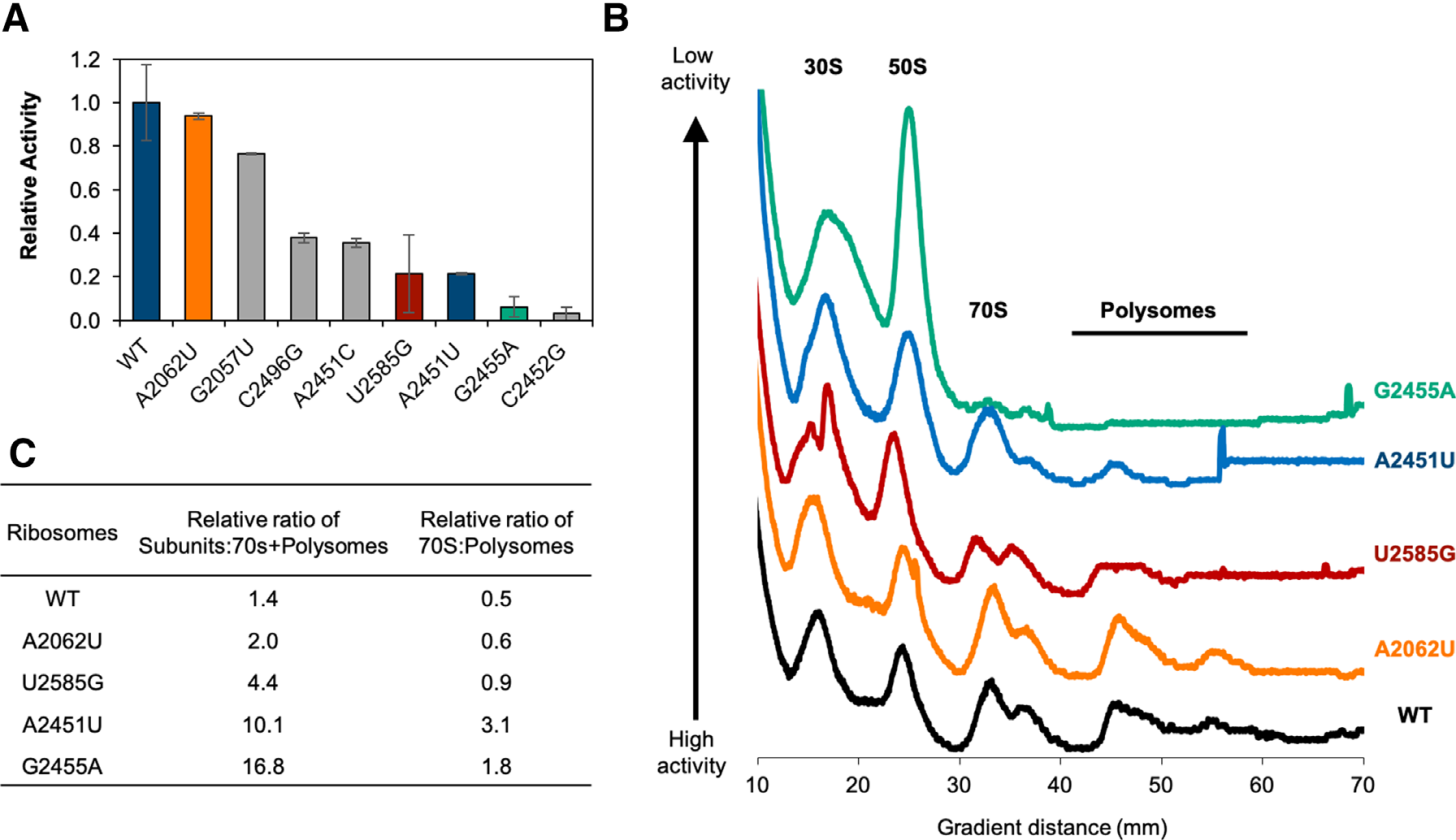
Sucrose gradient fractionation identifies assembly differences across ribosomal PTC mutants. (**A**) Five individual ribosome mutants were chosen for sucrose gradient fractionation based on their activity. One high activity (A2062U) mutant was chosen, two medium activity (U2585G and A2451U), and one low activity mutant (G2455A) was assessed for assembly and compared to WT. (**B**) A wild-type sucrose gradient fractionation trace (black) is compared to 4 representative mutant traces (color coded based on nucleotide mutation activity graph). From bottom to top, the mutants are positioned in decreasing activity order. (**C**) Relative areas under sucrose gradient fractionation trace curves were used to calculate ratios of subunits to 70S and polysome particles as well as the ratio of 70S particles to polysomes.

For all the PTC mutants, but especially those with low activity, we wondered if activity was related to the mutants’ ability to assemble into functional 70S ribosomes and translate in polysomes. This is because iSAT combines ribosome assembly and translation in a single-pot reaction. It is possible that an rRNA mutation may impact assembly (as opposed to molecular function), resulting in reduced translation activity. To this point, we analyzed assembly of a subset of mutant ribosomes by observing the 30S subunit, 50S subunit, 70S particles and polysomes (disomes and trisomes) using sucrose gradient fractionation as previously described (43) (Figure 4). Using a sucrose gradient, iSAT reactions were centrifuged, fractionated, and trace peaks analyzed (Supplementary Table S4). A set of high-, medium-, and low-activity mutants were chosen for analysis, with a few mutants also possessing compromised translation fidelity (U2585G, and A2451U). In the PTC-ring, all mutants except for G2455A broadly possessed similar assembly profile traces to wild-type iSAT ribosomes (Figure 4B), but exhibited reduced populations of 70S species and polysomes (disomes and trisomes), matching their decreasing activity (Supplementary Table S4). Of note, G2455A, exhibited a very different profile, accumulating almost entirely as free 30S and 50S subunits and only 4% 70S species, 2% disomes and no trisomes (Supplementary Table S4).

Notably, upon analyzing the relative abundance of species, we observed that compared to wild-type—which has a relative ratio of subunits to 70S + polysomes value of ∼1.4—G2455A has an ∼11-fold greater population of individual subunits (relative ratio of G2455A subunits to 70S + polysomes is ∼16.8) (Figure 4C). These results closely mirror the stark difference in translation activity as compared to wild-type. When comparing the relative ratio of 70S to polysomes, wild-type and the mutant with highest activity (A2062U) have similar ratios (70S:polysomes values of 0.5 to 0.6). As activity decreases, this ratio tends to increase roughly proportionately, suggesting that fewer 70S ribosomes are accumulating as polysomes.

### The ribosome's active site is composed of high- and low-flexibility pockets and shells

We next set out to map our analysis of mutational flexibility, translational readthrough, and ribosome assembly onto the ribosome's 3D structure, which would facilitate understanding of the PTC active site. Toward this goal, we first wanted to gain insight into how proximity to tRNA molecules impacts mutational flexibility. We measured distances from A76 of the A- or P-site tRNAs to the average geometric center of each nucleotide (Supplementary Table S5 and S6). We then organized the nucleotides in order of closest to furthest from the P-site tRNA (and compared to distances from the A-site tRNA). Upon generating a one-dimensional heat map (Supplementary Figure S8), we find different patterns in the PTC-ring compared to the A- and P-loop. Specifically, we found that the PTC-ring possesses pockets of high mutational flexibility (dark orange) and low mutational flexibility (white) regardless of distance from tRNA molecules. Whereas in the A- and P-loops there exists a more evident gradient of flexibility and activity, with the nucleotides residing closest to the P-site tRNA (4 Å) having the least amount of activity upon mutation, and the nucleotides residing furthest from the P-site tRNA (36 Å) having the greatest mutant activity.

**Figure 5.**
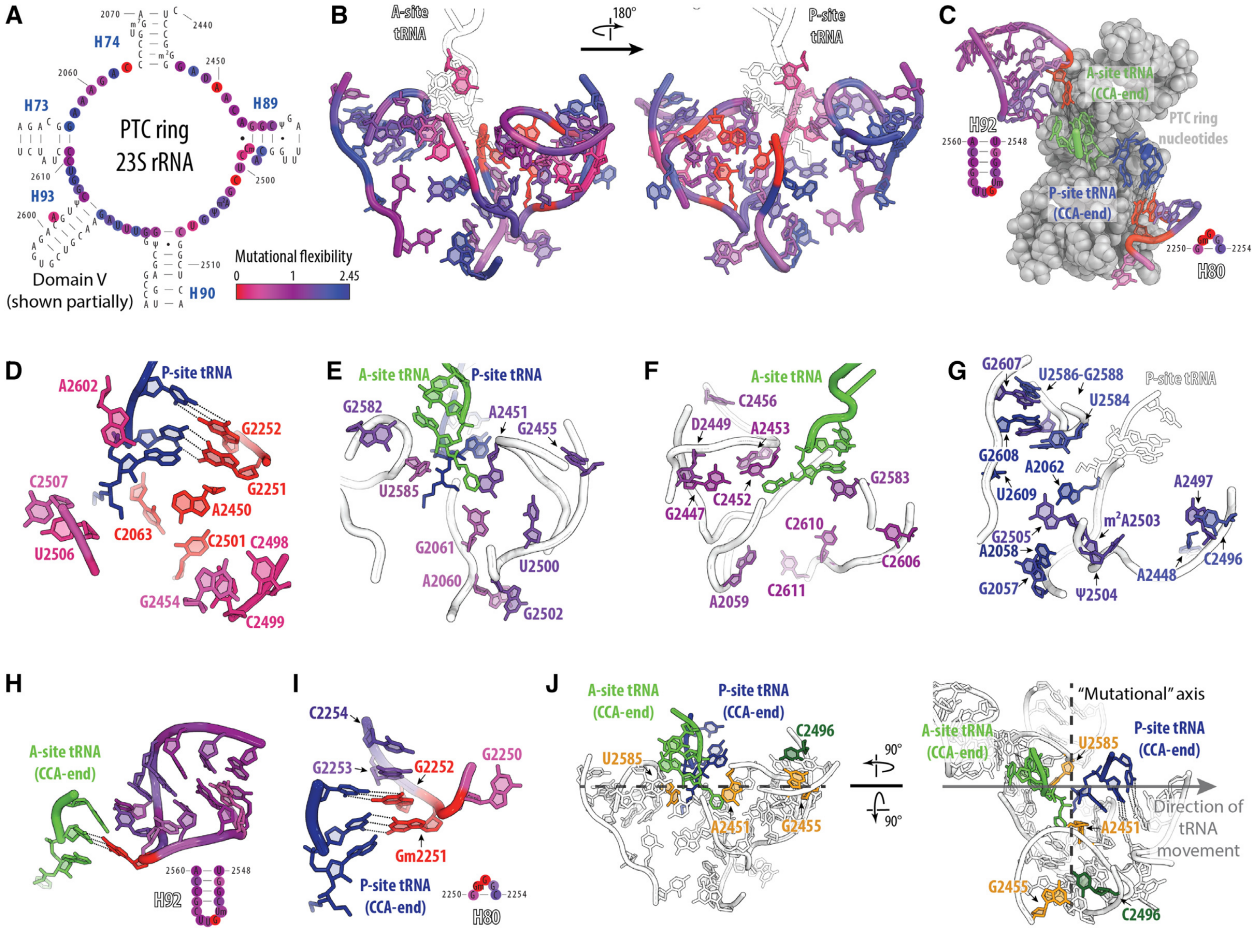
A mutational map reveals that the ribosome's PTC is composed of functional pockets and shells. (**A**) Secondary structure and (**B**) crystal structure model of the PTC-ring nucleotides probed in this study (heat mapped), along with the A-site tRNA, and P-site tRNA. (**C**) Crystal structure and secondary structure models of the A- and P-loop nucleotides probed in this study (heat mapped), A-site tRNA (green), P-site tRNA (blue), and PTC ring nucleotides (grey). (D–J) Crystal structure model of the PTC-ring nucleotides possessing: (**D**) the lowest mutational flexibility (red and magenta), (**E**) medium/low mutational flexibility (red-violet), (**F**) medium/high mutational flexibility (violet), and (**G**) the highest mutational flexibility (violet-blue). (**H**) Crystal structure model of the A-loop nucleotides probed in this study (heat mapped), A-site tRNA (green). (**I**) Crystal structure model of the P-loop nucleotides probed in this study (heat mapped), P-site tRNA (blue). (**J**) Structure model highlighting the nucleotides with mutants possessing increased translation readthrough (C2496, U2585 and A2451), as well as assembly defects (G2455). Ribosome structure accessed from PDB ID: 4YBB, with tRNAs from PDB ID: 1VY4.

By mapping our ribosome mutants’ activity onto the 3D ribosomal crystal structure, we then generated a mutational flexibility map of the active site (Figure 5 and Supplementary video). Upon deconvolution of the map into high-, medium- and low- mutational flexibility groups, we found mutationally flexible and inflexible shells, pockets, and a gradient of flexibility in the PTC-ring, A- and P-loops (Figure 5, Supplementary Figure S9 and Supplementary video). Furthermore, there were no major trends in maintaining high activity with nitrogenous base identity (purine vs pyrimidine) (Supplementary Figure S10). Within the PTC-ring, the first shell of nucleotides with the lowest mutational flexibility are shaded in red-magenta. Of these nucleotides, A2450, C2063 and C2501 possess the lowest mutational flexibility, and form a functionally critical pocket surrounding the P-site tRNA molecule with P-loop nucleotides G2252 and G2251 (Figure 5D) (65). In the next shell, nucleotides possessing medium/low mutational flexibility are shaded in red-violet, and on average reside in closer proximity to the P-site tRNA than the A-site tRNA. This nucleotide group includes G2455, which possesses assembly defects when mutated to G2455A, as well as U2585 and A2451, which possess increased translation readthrough when mutated to U2585G and A2451U, respectively (Figure 5E). The next shell of increasing mutational flexibility is shaded in violet. This shell of nucleotides spans both sides of the tRNA molecules and begins surrounding the exit tunnel (Figure 5F). Finally, within the shell of highest mutational flexibility (violet–blue), there resides a prominent pocket surrounding the exit tunnel. Of note, this shell houses the nucleotide C2496, which possesses high translation readthrough when mutated to C2496G (Figure 5G).

Within the A-loop nucleotides, there is a clear gradient of mutational flexibility, with the least flexible nucleotide being G2553 (red) and residing nearest to the A-site tRNA (4 Å). Importantly, this nucleotide makes key Watson–Crick interactions with the CCA-end (specifically C75) of the A-site tRNA (13) (Figure 5H), while nucleotides possessing high mutational flexibility (violet–blue) make minimal contacts with the A-site tRNA molecule (Figure 5C and H). Much like the A-loop nucleotides, the P-loop nucleotides also possess a gradient of mutational flexibility corresponding with distance from the CCA-end of the P-site tRNA molecule. Importantly, the least mutationally flexible nucleotides, G2252 and G2251, make key Watson–Crick base pairing interactions with C75 and C76, respectively (Figure 5C and I). Interestingly, when modelled onto the heat map of the PTC-ring nucleotides with the lowest mutational flexibility, there is a clear pocket of translationally critical nucleotides that begin with Watson-Crick base pairing interactions at C75 and stretch down to the attached amino acid (Figure 5D). Finally, for the A- and P-loops, we also analyzed regression models of distance from A-site and P-site tRNAs against mutational flexibility of A- (red) and P-loop (blue) nucleotides (Supplementary Figure S9). The regression plots for the A-loop nucleotides possess *R*^2^ values of 0.35 (*P* = 0.03) and 0.32 (*P* = 0.04), respectively. The regression plots for the P-loop nucleotides possess *R*^2^ values of 0.61 (*P* = 0.12) and 0.43 (*P* = 0.23), respectively. The regressions and *P*-values for the A-site nucleotides suggests a significant and predictive relationship between mutational flexibility and distance from tRNA molecules; while the P-site nucleotides suggests a predictive relationship, however this relationship is non-significant due to a small sample size.

We next combined our mutational flexibility maps with knowledge from the translation readthrough and assembly experiments. Upon analyzing the PTC-ring nucleotides with translation readthrough defects (C2496, U2585 and A2451), our mutational map highlights their unique positioning along the tRNA path through the ribosome (Figure 5J). Additionally, G2455, which possesses an assembly defect, resides just behind the A-site tRNA molecule. Finally, the nucleotides with the highest (violet-blue) mutational flexibility and lowest (red-magenta) mutational flexibility, reside in pockets that span opposite sides of the tRNA molecules (Figure 5D and G). Upon analyzing regressions for the PTC-ring nucleotides’ mutational flexibility against distance from each tRNA, we found no significant relationships (Distance from A-site tRNA: *R*^2^ = 0.154, *P* = 0.13; Distance from P-site tRNA: *R*^2^ = 0.001, *P* = 0.93), indicating that nucleotide distance alone does not explain the observed variation in mutational flexibility within this loop (Supplementary Figure S9).

## DISCUSSION

Here, using the iSAT platform, we systematically designed, built, and characterized 180 single point mutations within the ribosome's active site; specifically, targeting rRNA nucleotide positions that have been considered essential to peptide bond formation. Importantly, we have demonstrated that our results: (i) corroborate and build upon previous biochemical and structural studies; (ii) provide a comprehensive mutational map that may benefit future synthetic biology and engineering applications and (iii) provide mechanistic insight into peptide bond formation.

When comparing our results to previously published works, we find that our data fit well into early biochemical and structural studies; validating the power and accuracy of the iSAT system. For example, when looking at translation activity, our results align closely with others. In their 1996 study, Porse *et al.* assayed rRNA mutants for peptidyl transferase activity *in vitro* using a fragment assay (66). They found that upon mutating U2585 to U2585G, this mutation retained 36% of its peptidyl transferase activity (21% activity in our work) whereas U2585A and U2585C were <6% active (∼2% activity in our work). Furthermore, the authors found that G2253A, G2253U and G2253C carried 19%, 42% and <5% *in vitro* activity, respectively (in the same order: 55%, 40% and 14% activity in our work). Additional mutants in their study possess activities comparable to ours. In separate work conducted by Thompson and colleagues, the authors analyzed mutations at nucleotides A2451 and G2447. Upon characterizing peptidyl transferase activity, the authors found A2451C decreases the rate of reaction ∼3-fold. In our work, the protein synthesis activity of A2451C is reduced 2-fold (23).

Additionally, our results corroborate previous readthrough studies as well. Specifically, we demonstrate impaired readthrough of A2451C, A2451U, C2496G and U2585G mutants. These results are consistent with published findings demonstrating that mutations of U2585 lead to a decrease in rate of factor-catalyzed peptide release (24,61). Thompson and colleagues also demonstrated that when probing translation readthrough A2451C and A2451U exhibited increased readthrough of a UGA premature stop codon ∼2-fold (23). Similarly, our work shows UGA stop codon readthrough of ∼1-fold and ∼2-fold for A2451C, and A2451U, respectively.

Upon comparing our assembly profiles to those in the literature, we also find several parallels. First, our assembly assay results confirm that rRNA possessing base changes are still capable of forming functional particles for protein synthesis (23); but also highlight mutant G2455A as possessing defects in maintaining a fully assembled 70S structure (including polysomes). More specifically, upon assaying incorporation of the mutated 23S rRNA into ribosomal particles, Porse and colleagues found that U2585G possessed a greater fraction of 50S subunits compared to 70S species, which we found in this work as well (Supplementary Table S4) (66). When Thompson and colleagues analyzed mutations at nucleotides A2451 and G2447, the authors found that A2451U assembled into 70S particles and accumulated in polysomes, however, at decreased levels compared to wild-type—mirroring our results (23,67,68).

Lastly, across the literature, there are commonly used antibiotic resistance mutations within the ribosome. A prime example is at positions A2062 and A2058. A2062U and A2058U confer macrolide resistance in *E. coli* and other bacteria (69). We would expect that if our results match the mutants’ activity in the cell, that these well-studied PTC mutations would have high or almost wild-type activity. Indeed, in our results, we found that A2062U and A2058U possess 94% and 84% of wild-type activity, placing these nucleotides in the shell of ‘highly mutationally flexible nucleotides’ on our map. Importantly, the published results presented here align well with our iSAT activity results; confirming that our platform is robust and generates assembled *E. coli* ribosomes with function that closely mimics that of the cell.

In addition to corroborating previous studies, our work has also resulted in a comprehensive mutational flexibility and characterization map of the ribosome's active site, the first to our knowledge. Our map supports the current mechanism of peptidyltransferase activity, which has been extensively studied. For example, it highlights (in red) the essential role of G2553, G2252 and G2251, which are known to position tRNA molecules for peptidyl transfer (7,27,70), and the dependence of faithful hydrogen bonding and wobble pairing within the triple-base pocket C2501·A2450·C2063 (65) (Figure 5D). Our findings additionally corroborate and elaborate on other findings that aimed to pinpoint a more specific mechanism for the role of these bases. For instance, in their 2010 paper, Chirkova *et al.* found that modifications at positions A2450–C2063, impair translation activity, while having little impact on peptide bond formation, tRNA drop-off, or ribosome-dependent EF-G GTPase activity. Together, these data support the hypothesis that the wobble pair between A2450 and C2063 plays a critical role in tRNA translocation through the PTC (71). Similarly, Bayfield *et al.* found that the A2450–C2063 wobble pair do not play a direct role in peptidyl transferase activity, rather, they may function to maintain the correct structure of the active site in the context of the identity of other residues in the vicinity (72). Beyond known mechanistic understanding, our map also illustrates new insights into sequence-function relationships of rRNA nucleotides in the PTC. For example, we show the flexibility and the dispensable nature of nucleotides surrounding the exit tunnel (e.g. C2496, A2497, U2586, G2588, A2062, etc.), offering new questions regarding the evolution and necessity of these positions (Figure 5G). Finally, our map also demonstrates the complexity of rRNA loop arrangements within the ribosome. Our results indicate that a nucleotide's mutational flexibility, or dispensability, can be dependent on its position with respect to tRNA molecules (A- and P-loop flexibility gradients), or simply to neighboring nucleotides (PTC-ring mutational pockets and shells) (Figure 5A–C and J). Taken together, our results show that many active site mutated ribosomes can faithfully carry out protein synthesis, implying that these conserved nucleotides are not strictly indispensable for ribosome-catalyzed peptide bond formation.

Finally, when extrapolating our findings to better understand the chemistry and mechanism of peptide bond formation, our work not only corroborates previous findings, but also illuminates new results for mutations that were not previously well-studied. First, our work confirms previous hypotheses that PTC-ring nucleotides may play a key role in release factor binding and peptide release (73), and thus mutating these positions may alter these functions. However, the work presented here also adds a new PTC-ring nucleotide mutation to this growing list: C2496G; which currently lacks, to our knowledge, functional and mutational studies (Supplementary Table S1). On the other hand, the juxtaposed A- and P-loops, which displayed no impact on translation readthrough, do not appear to be implicated in release factor binding or hydrolysis. Instead, these nucleotides may play a role in tRNA selection by the ribosome. Specifically, upon aminoacyl-tRNA release from elongation factor-Tu, the A-loop may aid in accommodation of aminoacyl-tRNA into the A-site, permitting subsequent peptide-bond formation (74). Thus, binding of tRNA by the A-loop may act as a 50S checkpoint coupled to accommodation in the small subunit's decoding center. Although our assays alone are not capable of discriminating between translation fidelity and termination but rather characterize them together; they suggest supporting key roles for these nucleotides in substrate positioning and release. Efforts to identify the molecular mechanism by which mutations in the large subunit incur miscoding are not well-understood. Although the small subunit is largely recognized as the site of decoding, previous studies have identified decoding changes upon mutating the large subunit (51,53,62,75). It is hypothesized that the arrangement and geometry of the tRNAs in elongating ribosomes is perturbed by these active site mutations, thus decreasing the rate of peptidyl transfer and promoting errors in mRNA decoding (7,8,53,62,76). Looking forward for synthetic biology applications, additional tests should be performed that assay missense and nonsense fidelity, such as those previously described (77).

In summary, the *in vitro* iSAT platform allowed us to rapidly produce (in hours) and study populations of mutant *E. coli* ribosomes without contamination of wild-type species or cell-viability constraints (45). In our first step towards characterizing these mutations, we discovered that despite the high degree of conservation within the ribosome's active site (∼75% of the nucleotide positions are 100% conserved), >85% of the rRNA PTC nucleotides are still mutationally flexible to a variety of base changes. Of note, we found that sequence conservation alone was a poor proxy for predicting mutational flexibility (Supplementary Figure S5). Next, we employed a premature stop codon assay to study readthrough impairments, which could be caused by decreased translation accuracy or release factor fidelity (decreased binding affinity or hydrolysis). We observed increased levels of translation readthrough with four unique PTC-ring mutants (C2496G, A2451C, U2585G and A2451U), but no translation readthrough across a subset of A- and P-loop mutants (C2559A, C2551A, U2552G, C2559A, C2551A and U2552G). In our final functional assay, we assessed ribosome assembly across a suite of high-, medium- and low-activity mutants with sucrose gradient fractionation. The results of our assay are consistent with the presence of lower activity mutant ribosomes possessing reduced populations of 70S and polysomes. In particular, G2455A possessed primarily 30S and 50S subunits, and almost no functional 70S or polysomes.

Looking forward, we anticipate that our work will inform design strategies to engineer mutant ribosomes for novel purposes (29,78–85). Whether the engineering involves expanding the ribosome's exit tunnel (most mutationally flexible and dispensable) or co-evolving nucleotide pockets that appear to rely on key hydrogen bonding and base-pairing (the most mutationally inflexible nucleotides), our new systems-level understanding is expected to help guide ribosome re-design by highlighting bases permissible to change (86). This in turn will increase our understanding the process of translation to advance new applications.

## DATA AVAILABILITY

The Protein Data Bank (PDB) is an open source archive containing information about experimentally-determined structures of proteins, nucleic acids, and complex assemblies (https://www.rcsb.org/pdb/home/home.do).

Atomic coordinates and structure factors for the ribosome crystal structure used in the described analyses can be found within the Protein Data bank under accession number 4YBB (87) and 1VY4 (tRNA molecules) (57).

PyMol is a computer software, a molecular visualization system (https://pymol.org/2/).

Code used for sequence aligning, sequence matrix visualization and entropy plotting is open source and available in the GitHub repository (https://github.com/adamhockenberry/23s-alignment-LTP).

## Supplementary Material

gkaa001_Supplemental_Files
