# Supplementary material for: Mutational characterization and mapping of the 70S ribosome active site
Source: Nucleic Acids Res. 2020 Feb 3;48(5):2777–89. doi: 10.1093/nar/gkaa001 (PMC7049736; doi:10.1093/nar/gkaa001)
Supplement: gkaa001_Supplemental_Files [file gkaa001_supplemental_files.zip › 2019-AED-PTC_Revised_Supplementary_File_NAR_Final.docx]

**Mutational characterization and mapping of the 70S ribosome active site**

Anne E. d’Aquino^1,2,3,4^, Tasfia Azim^2,3, 4^, Nikolay A. Aleksashin^4^, Adam J. Hockenberry^1,2,3,4^, Antje Krüger^2,3,4^, Michael C. Jewett^1,2,3,6,7*^

^1^ Interdisciplinary Biological Sciences Program, Northwestern University, Evanston, IL, 60208, USA
^2^ Department of Chemical and Biological Engineering, Northwestern University, Evanston, IL, 60208, USA
^3^ Chemistry of Life Processes Institute, Northwestern University, Evanston, IL, 60208, USA
^4^ Center for Synthetic Biology, Northwestern University, Evanston, IL, 60208, USA
^5^ Center for Pharmaceutical Biotechnology, University of Illinois at Chicago, Chicago, IL, 60607, USA
^6^ Robert H. Lurie Comprehensive Cancer Center, Northwestern University, Chicago, IL 60611, USA ^7^ Simpson Querrey Institute, Northwestern University, Chicago, IL 60611, USA

* To whom correspondence should be addressed. Tel: (847) 467-5007; Fax: (847) 491-3728; Email:
[m-jewett@northwestern.edu](mailto:m-jewett@northwestern.edu)

**Supplemental Information**

**
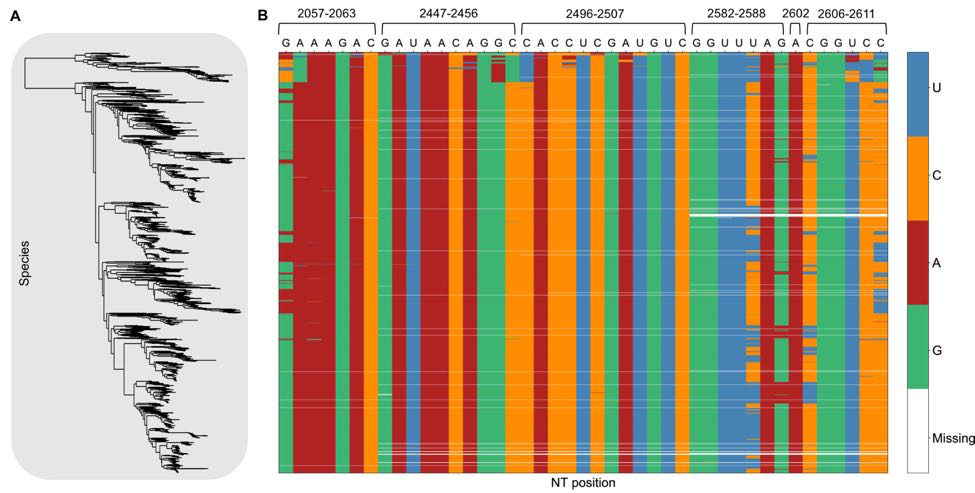
**

**Supplementary Figure S1. The ribosome’s peptidyl transferase center (PTC) is highly conserved. (A)** Phylogenetic tree of the species used for the rRNA sequence alignment. The left to right distance for any branch of the tree represents the amount of sequence divergence from the "root" (far left) to the actual sequence (the leaves, far right). **(B)** Sequence alignment matrix illustrating the conservation of PTC nucleotides across 1,614 species of bacterial and archael species. All LSU sequences were taken from alignments found in the SILVA database (43). The ribosome’s PTC is highly conserved across both bacterial and archael species.

**
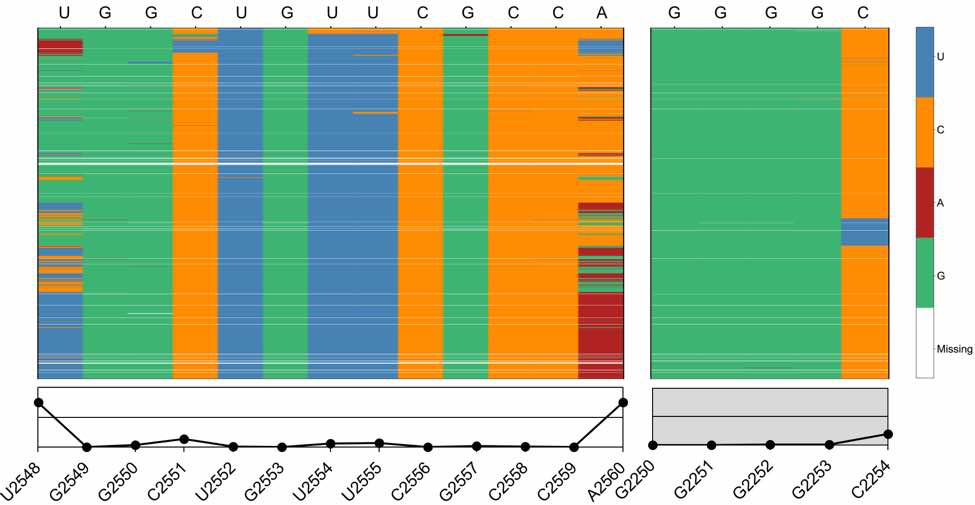
**

**Supplementary Figure S2. The ribosome’s A- and P-loops are highly conserved.** Sequence alignment illustrating the conservation of PTC nucleotides across 1,614 species of bacterial and archael species. All LSU sequences were taken from alignments found at <https://www.arb-silva.de/projects/living-tree/>. The ribosome’s PTC is highly conserved across both bacterial and archael species. Shannon Entropy scores are akin to variance scores (though we caution that they ignore phylogenetic relatedness), with a Shannon Entropy of zero representing zero variance (100% conservation across the 1,614 species). Any values above zero indicate that evolutionary changes have occurred and result in multiple nucleotides within a given site in the alignment.

**
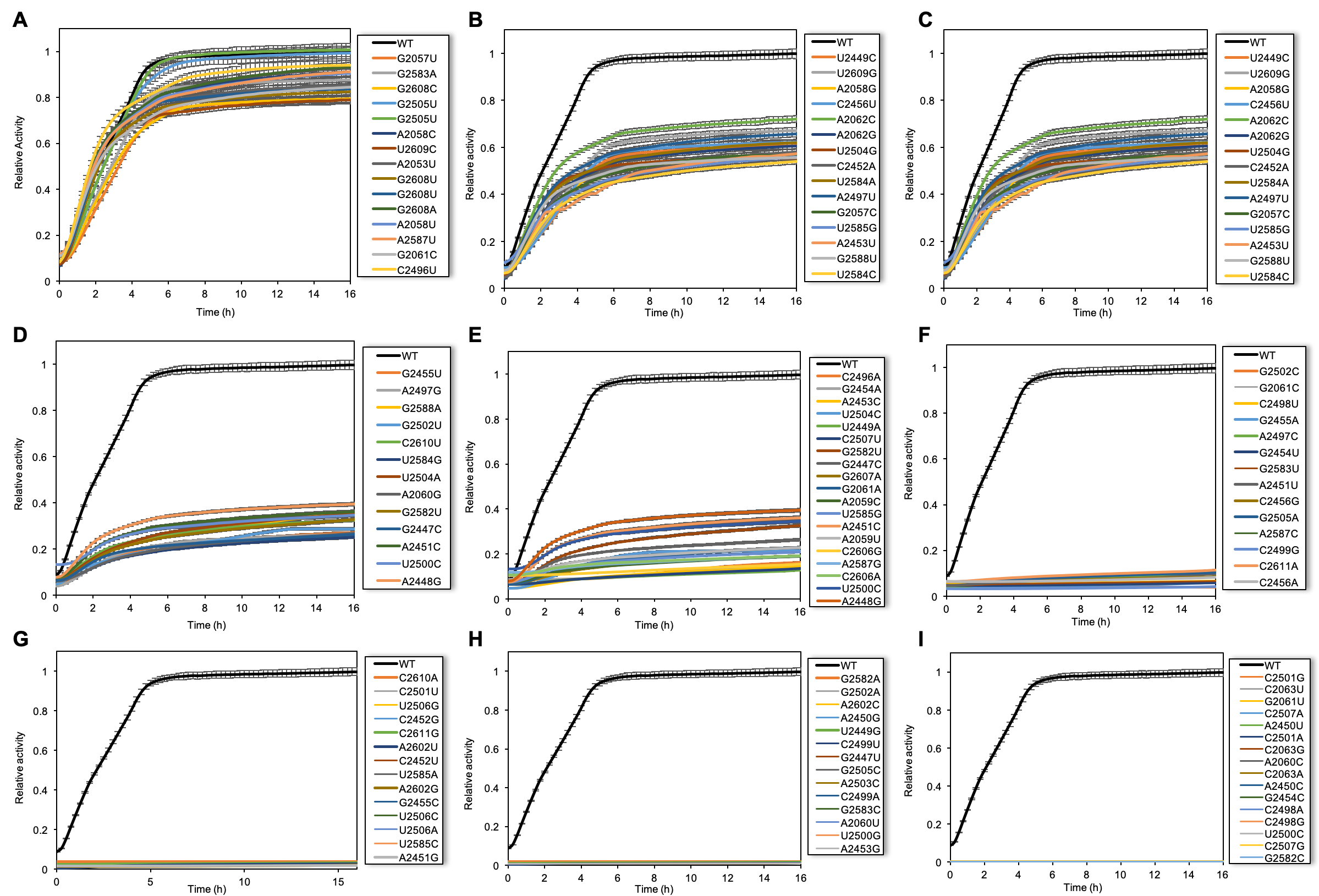
**

**Supplementary Figure S3.** Protein synthesis kinetic time course curves from iSAT reactions for all individual nucleotide mutations probed in the PTC-ring. (A-I) Data are arranged in groups of high-, medium-, and low-activity mutants. Smoothed data from samples taken every 15 minutes are shown. Values represent averages and error bars represent one standard deviation from the mean, with n ≥ 3 for n number of independent reactions.


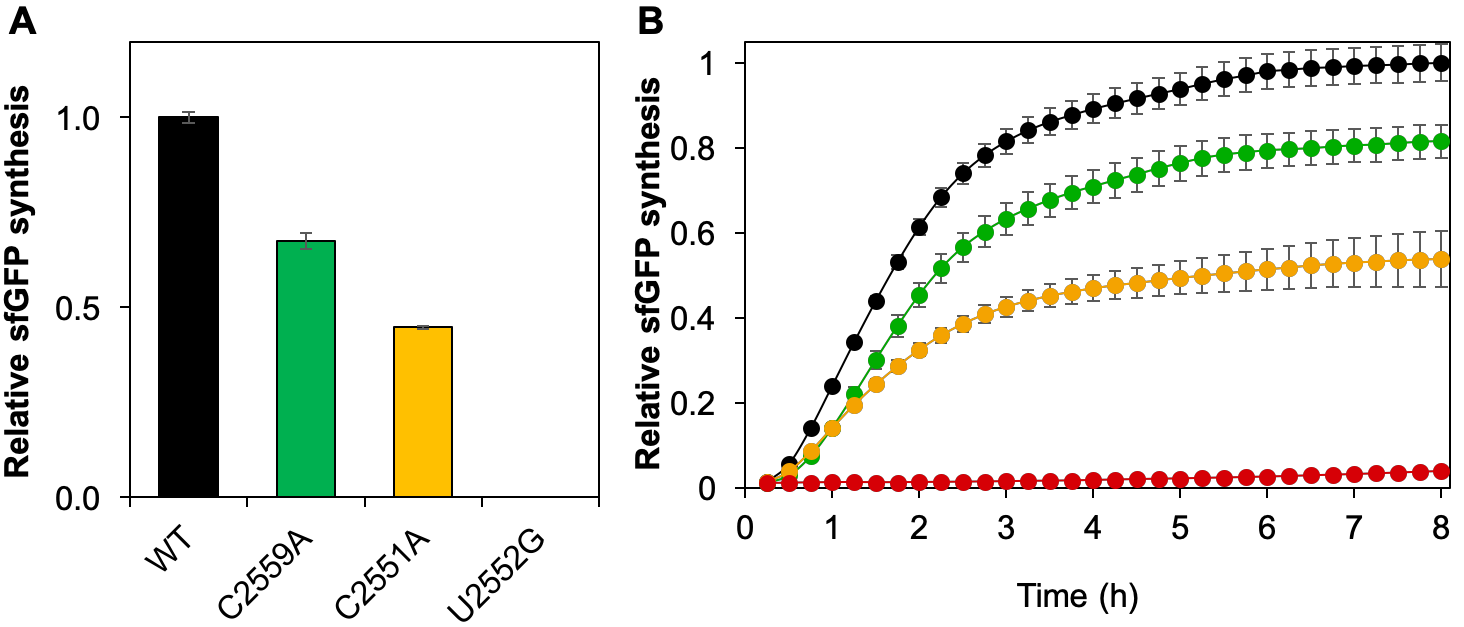


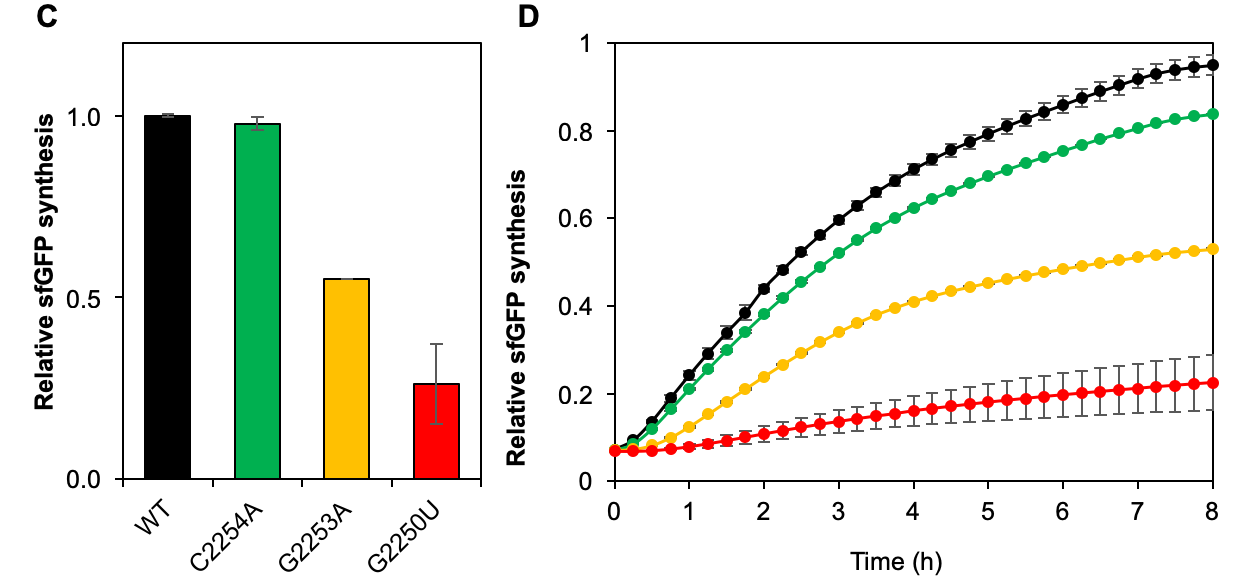


**Supplementary Figure S4. Ribosomes with mutations in the A- and P-loops demonstrate decreased bulk protein synthesis rates. (A)** Relative protein synthesis activity of A-loop mutants tested in translation assays. **(B)** Protein synthesis kinetic time course curves from iSAT reactions for the following A-loop nucleotide mutations have been included in this graph: C2559A, C2551A, U2552G. Protein synthesis rates are proportional to relative protein synthesis titers. For simplicity and ease of visualization, not all nucleotide mutation kinetic curves are included on the graph (see **Supplementary Figure S3**). Values represent averages and error bars represent one standard deviation from the mean, with n ≥ 3 for n number of independent reactions. **(C)** Relative protein synthesis activity of P-loop mutants tested in translation assays. **(D)** Protein synthesis kinetic time course curves from iSAT reactions for the following P-loop nucleotide mutations have been included in this graph: C2254A, G2253A, G2250U. Protein synthesis rates are proportional to relative protein synthesis titers. For simplicity and ease of visualization, not all nucleotide mutation kinetic curves are included on the graph (see **Supplementary Figure S3**). Values represent averages and error bars represent one standard deviation from the mean, with n ≥ 3 for n number of independent reactions.

**
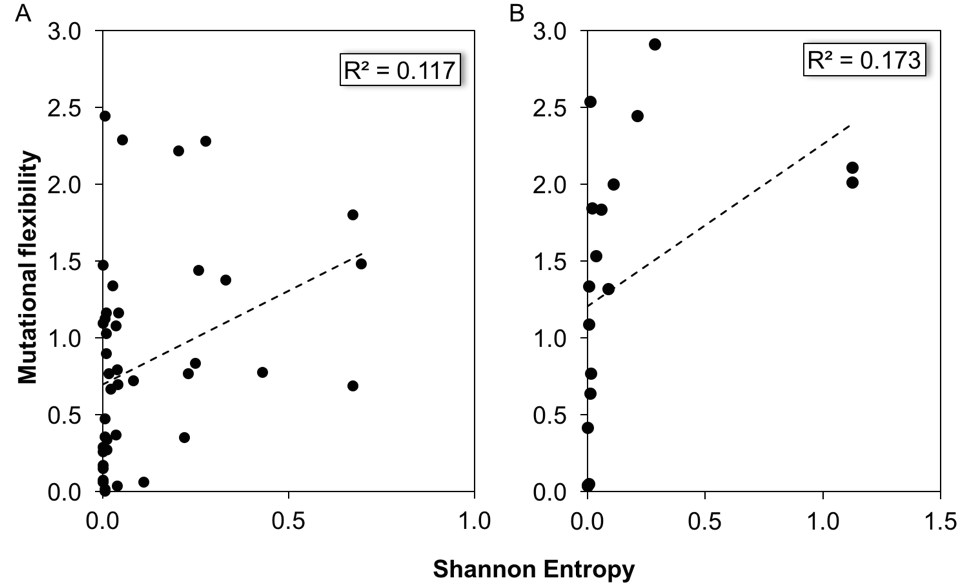
**

**Supplementary Figure S5. Regression models of Shannon entropy (nucleotide conservation) against mutational flexibility (the sum of all nucleotide mutations’ relative activity). (A)** The regression plot for the PTC-ring possesses a low R^2^ value (R^2^ =0.117, *p*=0.025) demonstrating difficulty in predicting mutational flexibility from nucleotide conservation. **(B)** The regression plot for the A- and P-loops also possesses a low R^2^ value (R^2^ =0.173, *p*=0.086) demonstrating difficulty in predicting mutational flexibility from nucleotide conservation.


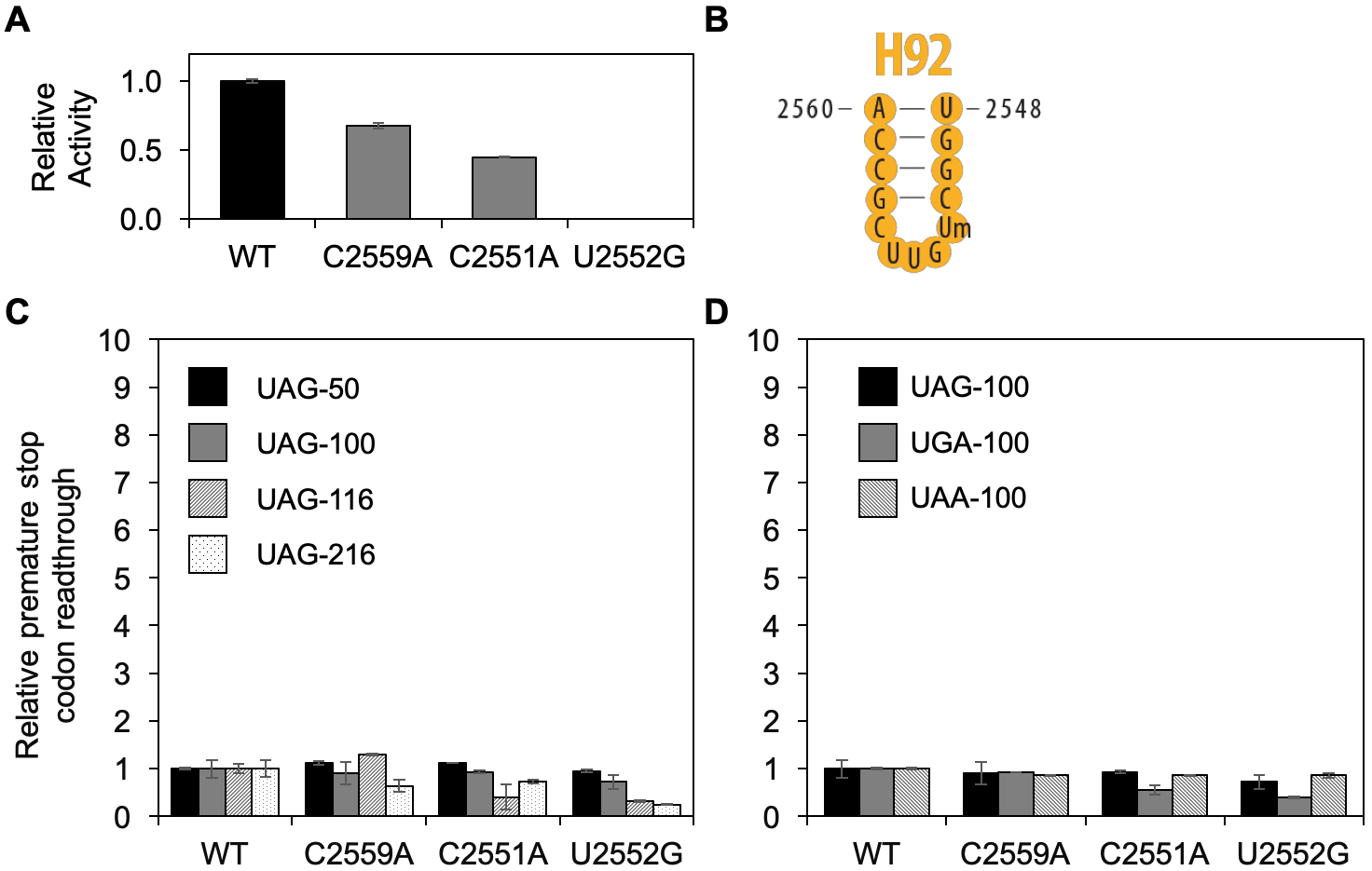


**Supplementary Figure S6. Ribosomal A-loop mutations do not increase stop-codon readthrough. (A)** Relative protein synthesis activity of A-loop mutants tested in translation fidelity assays. **(B)** A-loop secondary structure. **(C)** UAG stop codon readthrough at amino acid position 50, 100 ,116, and 216 of sfGFP. **(D)** UAG, UGA, and UAA stop codon readthrough at amino acid position 100 of sfGFP. As described in the main text, the relative readthrough activity in translation fidelity assays using premature stop codons was assessed using sfGFP fluorescence. sfGFP levels obtained with wild-type rRNA plasmids are normalized to 1, and values obtained with each of the mutants were expressed relative to that obtained with the respective wild-type rRNA plasmid. To enable comparison with Figure 3 from the main text, the y-axis values are extended to 10 in **C** and **D**. Values represent averages and error bars represent one standard deviation from the mean, with n ≥ 3 for n number of independent reactions.

**
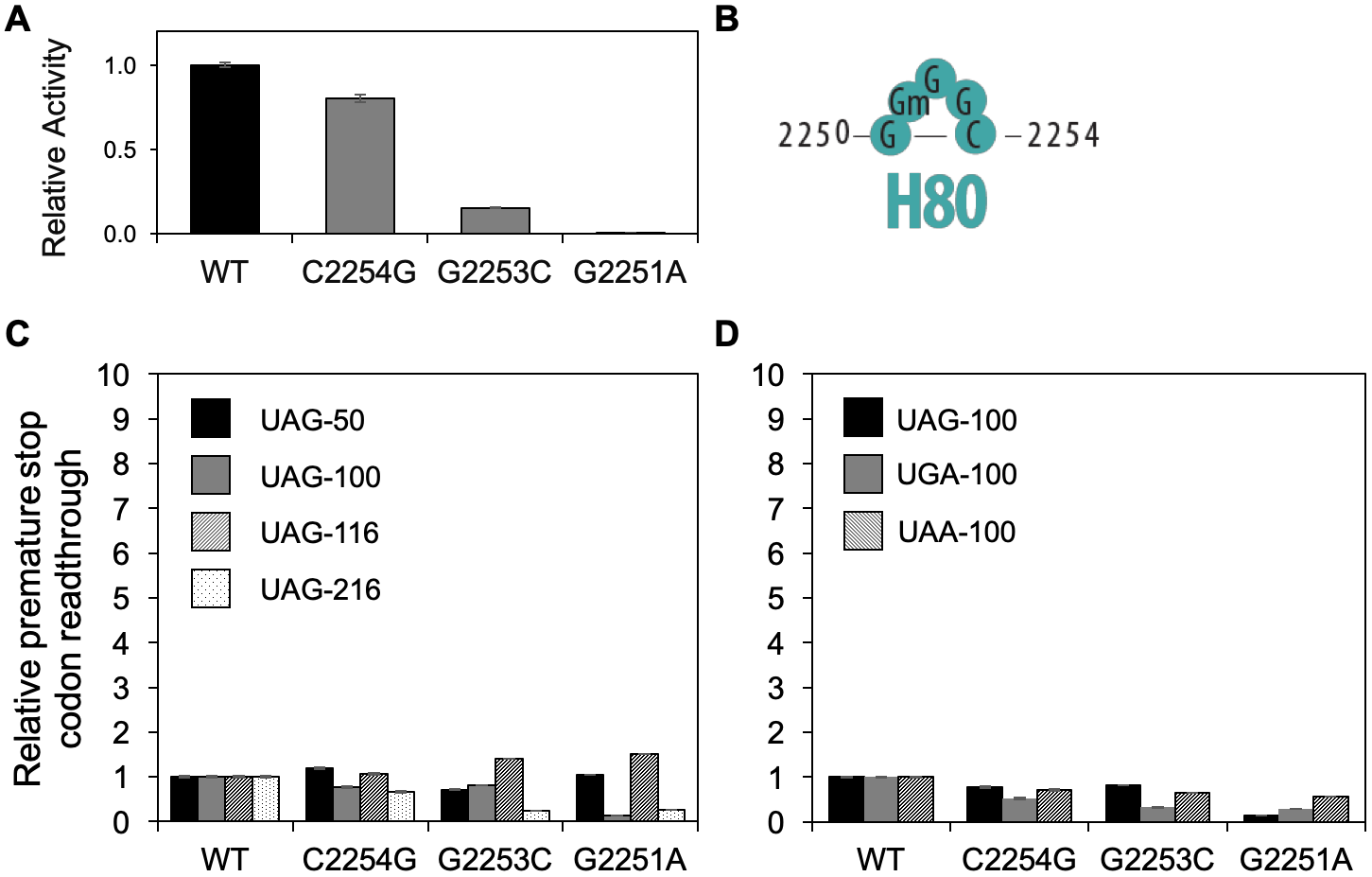
**

**Supplementary Figure S7. Ribosomal P-loop mutations do not increase stop-codon readthrough. (A)** Relative protein synthesis activity of P-loop mutants tested for fidelity. **(B)** P-loop secondary structure. **(C)** UAG stop codon readthrough at amino acid position 50, 100 ,116, and 216 of sfGFP. **(D)** UAG, UGA, and UAA stop codon readthrough at amino acid position 100 of sfGFP. As described in the main text, the relative readthrough activity in translation fidelity assays using premature stop codons was assessed using sfGFP fluorescence. sfGFP levels obtained with wild-type rRNA plasmids are normalized to 1, and values obtained with each of the mutants were expressed relative to that obtained with the respective wild-type rRNA plasmid. To enable comparison with Figure 3 from the main text, the y-axis values are extended to 10 in **C** and **D**. Values represent averages and error bars represent one standard deviation from the mean, with n ≥ 3 for n number of independent reactions.


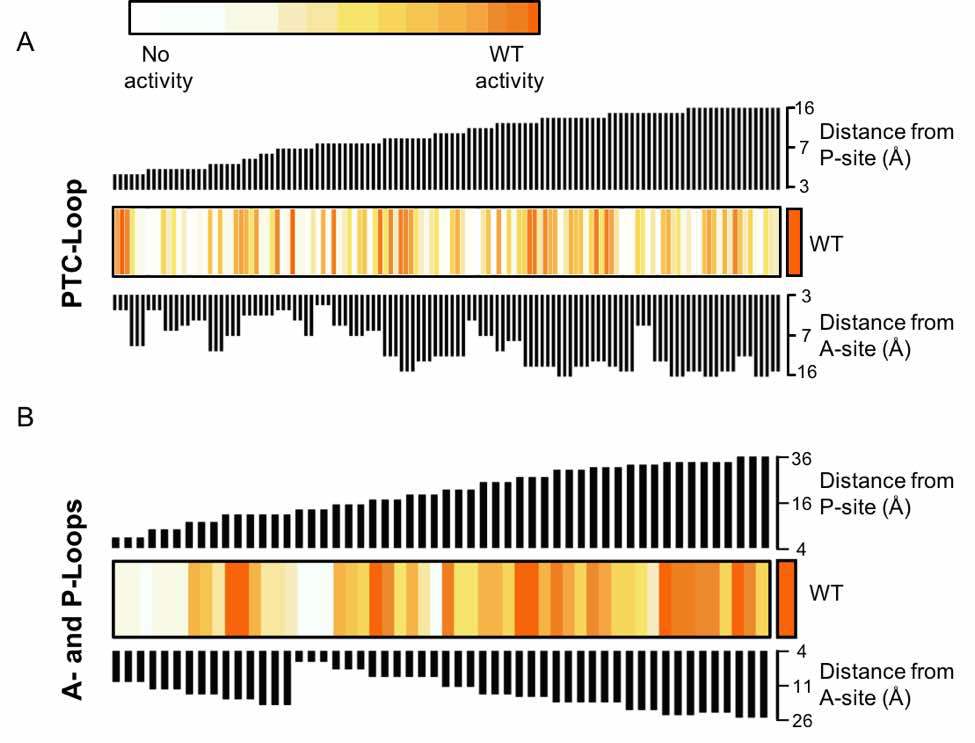


**Supplementary Figure S8.** **The ribosome’s peptidyl transferase center (PTC) is composed of functional pockets. (A)** Heat map of rRNA PTC-ring and **(B)** A- and P-loop mutational activity against their distance from the P-site tRNA (top) and A-site tRNA (bottom). White shading illustrates zero activity, while dark orange shading illustrates WT-activity.

**
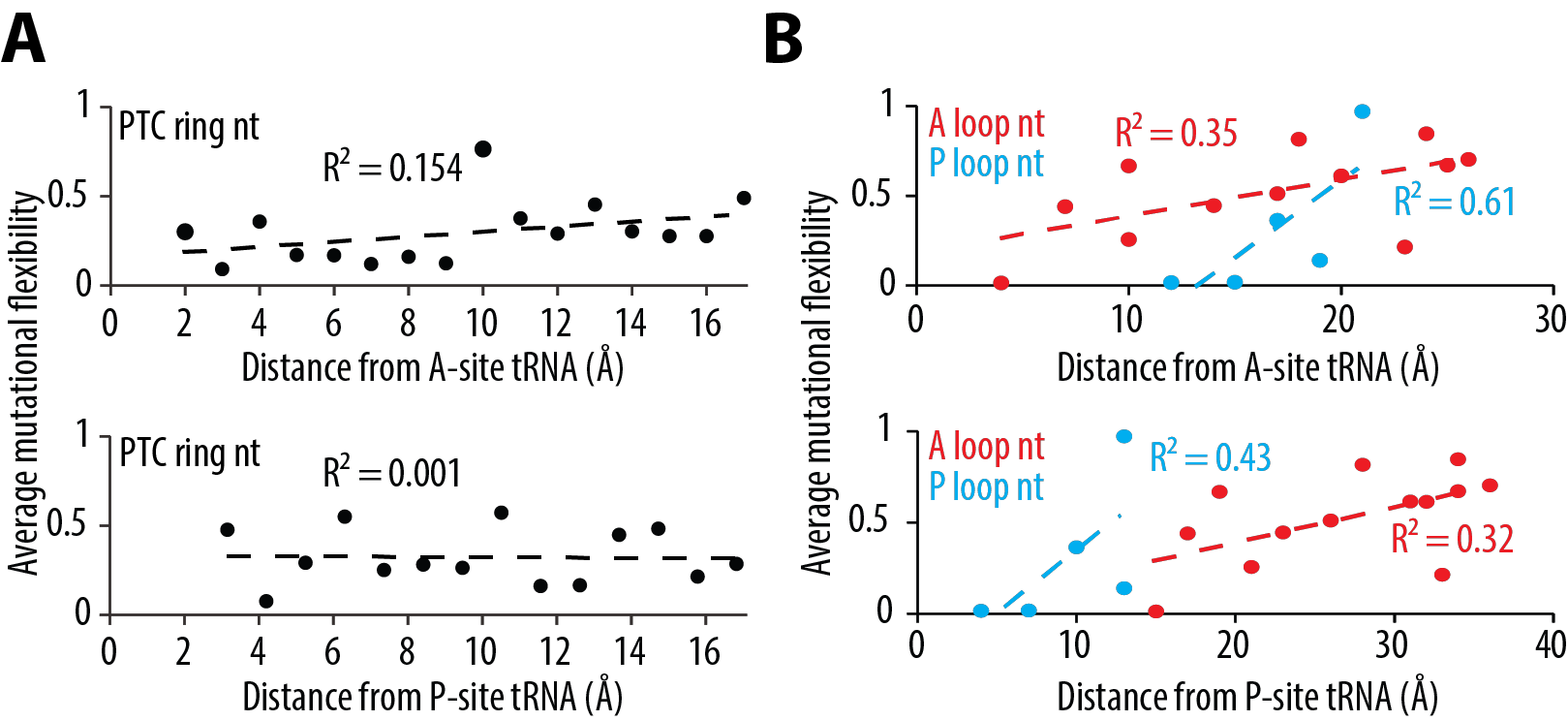
**

**Supplementary Figure S9. Regression models of nucleotide distance against mutational flexibility. (A)** Regression model of distance from A-site (R^2^=0.154, *p*=0.13) and P-site (R^2^=0.001, *p*=0.93) tRNA against mutational flexibility of PTC-ring nucleotides suggests a non-significant (*p*>0.05) and weak (low R^2^ values) relationship. **(B)** Regression model of distance from A-site and P-site tRNA against mutational flexibility of A- (red) and P-loop (blue) nucleotides. The regression plots for the A-loop nucleotides possess R^2^ values of 0.35 (*p*=0.03) and 0.32 (*p*=0.04), respectively. The regression plots for the P-loop nucleotides possess R^2^ values of 0.61 (*p*=0.12) and 0.43 (*p*=0.23), respectively. The regressions and *p*-values for the A-site nucleotides suggests a significant and predictive relationship between mutational flexibility and distance from tRNA molecules; while the P-site nucleotides suggests a predictive relationship, however this relationship is non-significant due to a small sample size.

**
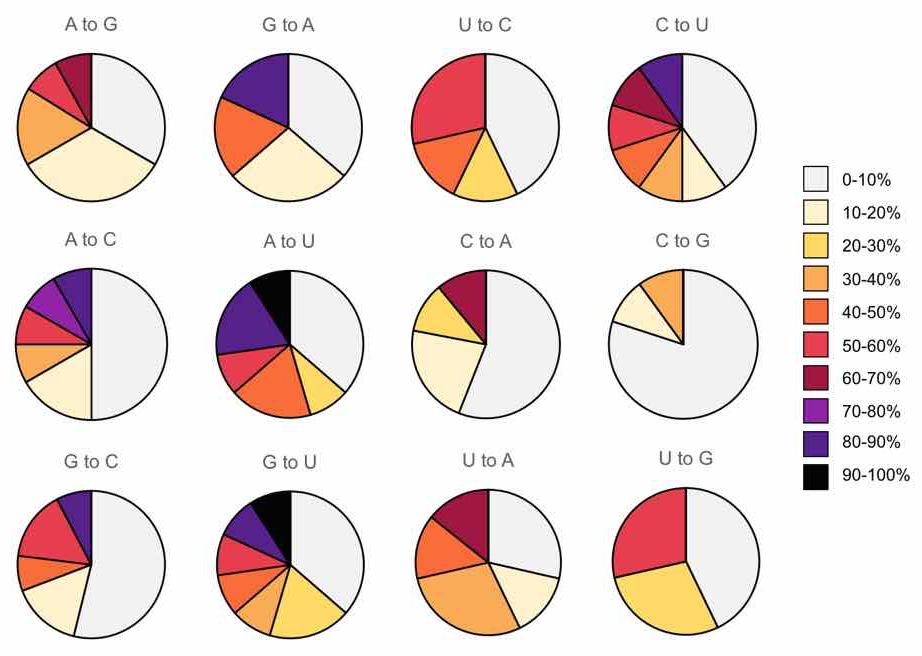
**

**Supplementary Figure S10. Comparison of ribosomal mutant activity vs. nucleotide base change within the PTC-ring.** Nucleotide changes from C to A, C to G, and G to C have the lowest activity, with ~70-90% of the nucleotides exhibiting less than 20% of WT activity. In contrast, changes from G to A, A to U and G to U exhibit the highest activity, with ~20-30% of the nucleotides exhibiting 80-100% of WT activity.

**
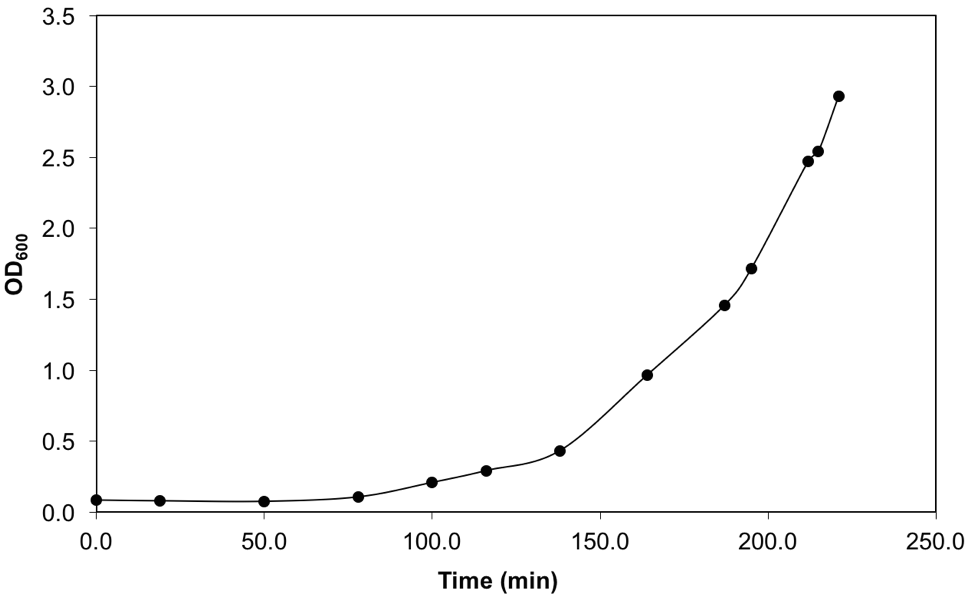
**

**Supplementary Figure S11.** Growth curve of E. coli MRE600 strain used in this study. Cells were grown in 10L of 2xYTPG media and harvested from the fermenter at OD = 2.9–3.1. Cells were grown at 37C, as previously reported.

**
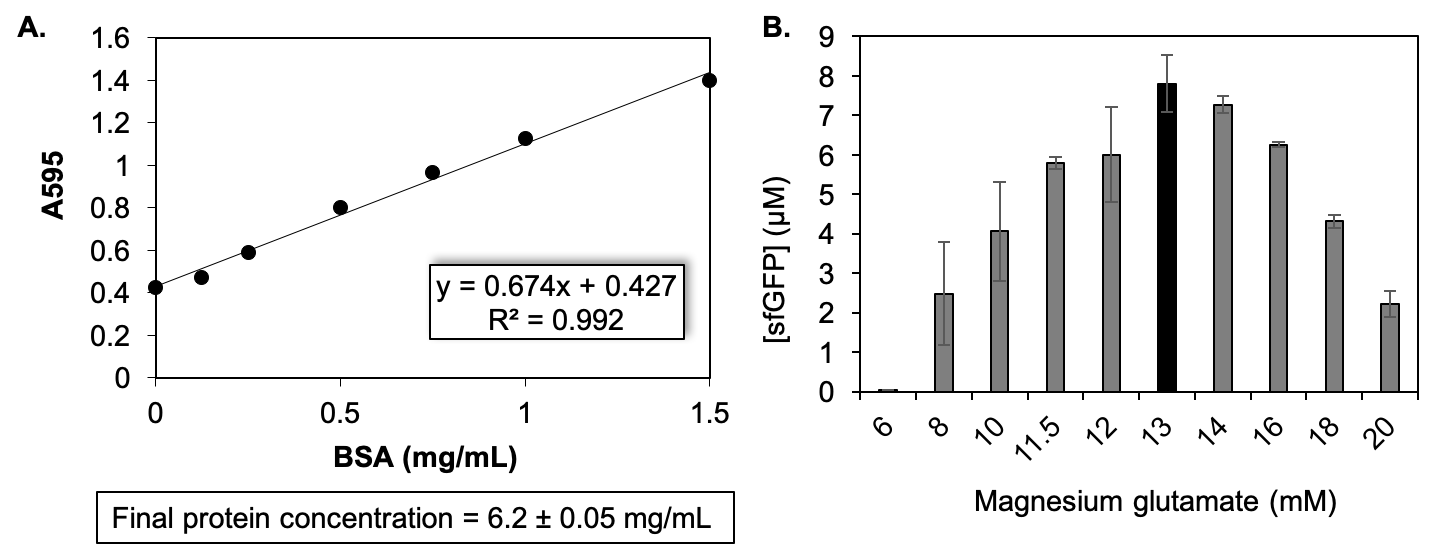
**

**Supplementary Figure S12. Extract testing and optimization. (A)** Protein concentration of S150 extract used in this paper. Values represent average concentrations as determined by Bradford assay with bovine serum albumin (BSA) as a standard. Error bars represent one standard deviation from the mean for triplicate measurements. **(B)** Magnesium optimization of S150 extract for reporter protein synthesis in iSAT reactions. Standard 15 μL batch reactions were performed at 37°C for 20 h, with varying magnesium glutamate concentrations the S150 extract used. Total protein concentration of each S150 extract added to reactions was standardized at 3.6 mg mL^-1^. Synthesis of active wild-type (wt) sfGFP was measured after 20 h using fluoresence. Optimal magnesium glutamate concentrations for iSAT reactions was determined to be: 13 mM for MRE600. This concentration was then used for subsequent experiments. Values represent averages and error bars represent one standard deviation from the mean, with n ≥ 3 for n number of independent reactions.


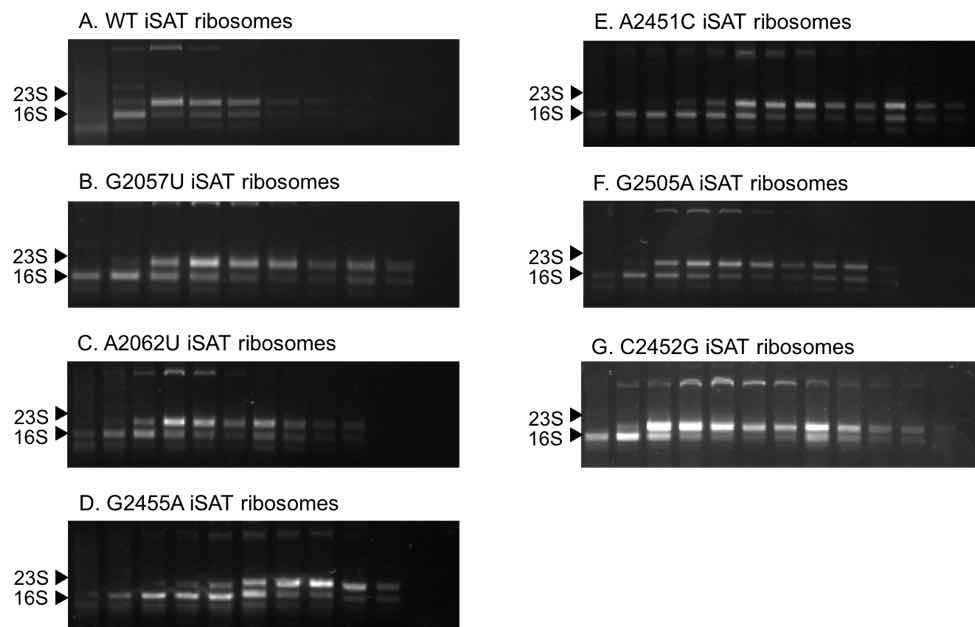


**Supplementary Figure S13. Gel electrophoresis analysis of WT and mutant iSAT ribosomes following sucrose density centrifugation.** A 120 μL iSAT reaction possessing a reporter plasmid was analyzed by polysome profiling in a 10-40% sucrose gradient. The resulting 500 μL – 1 mL fractions were run by electrophoresis on a 1% agarose gel (one fraction per well). The rRNA contained within each fraction was used to confirm the predominate peaks as containing 30S, 50S, or 70S subunits.

**Supplementary Table S1.** *E. coli* 23S rRNA PTC nucleotides and their published mutational studies.

| **rRNA Nucleotide** | **Mutational studies** | **Reference** |
| --- | --- | --- |
| **G2057** | - G2057A confers low-level resistance to erythromycin in *E. coli* - G2057C remains unstudied - G2057U remains unstudied | - Ettayebi, M., Prasad, S.M., and Morgan, E.A. J Bacteriol. 1985. 162(2): 551-557. |
| **A2058** | - A2058G confers macrolide resistance - A2058U confers clindamycin resistance - A2058C remains unstudied | - Xiong et al. Antimicrob. Agents Chemother. 2005. 49(1): 281-288. - Cochella & Green. PNAS. 2004. 101(11): 3786-3791. - Prunier, A., et al. Antimicrob Agents Chemother. 2002. 46(9): 3054-3056. - Pfister, P., et al. PNAS. 2005. 102(14) 5180-5. |
| **A2059** | - A2059G confers macrolide resistance in other bacteria (*E. coli* mutation unstudied) - A2059U remains unstudied - A2059C remains unstudied | - Poehlsgaard, J., et al. Antimicrob Agents Chemother. 2005. 49(4): 1553-1555. |
| **A2060** | - A2060G remains unstudied in *E. coli* (*M. bovis* exhibits tiamulin- and valnemulin resistance) - A2060U remains unstudied - A2060C remains unstudied due to lethality in vivo | - Sulyok, K. et al. 2017. Antimicrob Agents Chemother. 61(2): e-01983-16. - Vester, B., and Garrett, R. 1988. EMBO Jour. 7(11):3577-3587. |
| **G2061** | - G2061A remains unstudied in *E. coli* (antibiotic resistance in other bacteria – Pristinamycin resistant in *T. therophilus*) - G2061C remains unstudied in *E. coli* - G2061U remains unstudied in *E. coli* (tiamulin and valnemulin resistance in *M. gallisepticum* when in combination with other mutations; and clindamycin resistance in *T. gondii*) | - Gregory, S. et al. 2005. J. Bacteriol. 187(14): 4804-4812 - Li, B., et al. 2010. FEMS Microbiology letters. 308(2):144-149. - Camps, M., et al. Mol. Microbiol. 2002. 43(5): 1309-18. |
| **A2062** | - A2062G confers clindamycin resistance in *E. coli*. Confers macrolide resistance in other bacteria - A2062U confers macrolide resistance in other bacteria - A2062C remains unstudied in *E. coli*. Confers 16-memberd macrolide resistance in *S. pneumoniae*. | - Cochella & Green. PNAS. 2004. 101(11): 3786-3791. - Furneri, P., et al. 2001. Antimicrob Agents Chemother. 2001. 45(10): 2958-2960. - Depardieu, F., and Courvalin, P. 2001. Antimicrob Agents Chemother. 2001. 45(1):319-323. |
| **C2063** | - C2063A remains unstudied - C2063U only studied in the context of A2450G mutation. The combination of mutations decreases rate of peptide bond formation. - C2063G remains unstudied | - Hesslein, A.E., et al. NAR. 2004. 32(12): 3760-3770. |
| **G2447** | - G2447A resistant to oxazolidinones - G2447U dominant lethal in vivo - G2447C resistant to streptomycin, but slow growth. | - Babkova, E.V., et al. JBC. 2003. 278(11): 9802-9807. - Thompson, J., et al. PNAS. 2001. 98(16) 9002-9007. |
| **A2448** | - A2448C remains unstudied - A2448G remains unstudied - A2448U remains unstudied |  |
| **U2449** | - U2449A strongly dominant lethal in vivo - U2449G strongly dominant lethal in vivo - U2449C viable with no impact on growth rate | - Yassin, A.S. & Mankin, A.S. Journal of Biological Chemistry. 2007. 282(33): 24329-24342. - O’Connor, M., et al. NAR. 2001. 239(3): 710-5. |
| **A2450** | - A2450G strongly dominant lethal in vivo - A2450U remains unstudied - A2450C remains unstudied | - Yassin, A.S. & Mankin, A.S. Journal of Biological Chemistry. 2007. 282(33): 24329-24342. |
| **A2451** | - A2451U strongly dominant lethal in vivo - A2451G strongly dominant lethal in vivo. Deficient in early assembly steps of large subunit. - A2451C peptidyltransferase reaction rate reduced by ~4-fold | - Yassin, A.S. & Mankin, A.S. Journal of Biological Chemistry. 2007. 282(33): 24329-24342. - Thompson, J., et al. PNAS. 2001. 98(16): 9002-9007. |
| **C2452** | - C2452A remains unstudied - C2452U confers anisomycin resistance in *H. marismortui* and *S. cerevisiae* - C2452G remains unstudied | - Blaha, G., et al. J. Mol. Biol. 2008. 379(3): 505-519 |
| **A2453** | - A2453C confers resistance to anisomycin and to linezolid in *H. halobium*. Decreased growth rate. - A2453G strongly dominant lethal in vivo - A2453U confers resistance to anisomycin in *H. marismortui*. | - Kloss, P., et al. JMB. 1999. 294: 93-101. - Yassin, A.S. & Mankin, A.S. Journal of Biological Chemistry. 2007. 282(33): 24329-24342. - Blaha, G., et al. J. Mol. Biol. 2008. 379(3): 505-519 |
| **G2454** | - G2454A moderately dominant lethal in vivo - G2454U remains unstudied - G2454C remains unstudied | - Yassin, A.S. & Mankin, A.S. Journal of Biological Chemistry. 2007. 282(33): 24329-24342. |
| **G2455** | - G2455A remains unstudied - G2455U remains unstudied - G2455C remains unstudied |  |
| **C2456** | - C2456A remains unstudied - C2456U remains unstudied - C2456G remains unstudied |  |
| **C2496** | - C2496A remains unstudied - C2496U remains unstudied - C2496G remains unstudied |  |
| **A2497** | - A2497C remains unstudied - A2497G strongly defective in peptidyl transferase activity - A2497U remains unstudied | - Porse, B.T., and Garrett, R.A. JMB. 1995. 249, 1-10. |
| **C2498** | - C2498A remains unstudied - C2498U remains unstudied - C2498G remains unstudied |  |
| **C2499** | - C2499A alone or when paired with G2032A confers linezolid resistance - C2499U strongly dominant lethal in vivo - C2499G remains unstudied | - Fulle, S., et al. NAR. 2015 43(16): 7731-7743. - Yassin, A.S. & Mankin, A.S. Journal of Biological Chemistry. 2007. 282(33): 24329-24342. |
| **U2500** | - U2500A confers anisomycin resistance in *H. marismortui* - U2500G remains unstudied - U2500C confers anisomycin resistance in *H. marismortui*. Confers linezolid resistance in *E. coli* and *H. halobium*. | - Blaha, G., et al. J. Mol. Biol. 2008. 379(3): 505-519. - Xion, L.Q., et al. J. Bacteriol. 2000. 182: 5325-5331. |
| **C2501** | - C2501A remains unstudied - C2501U remains unstudied - C2501G remains unstudied |  |
| **G2502** | - G2502A causes a decreasesd growth rate in *E. coli.* - G2502U remains unstudied - G2502C remains unstudied | - Vester, B., and Garrett, R.A. EMBO J. 1988. 7(11): 3577-3587. |
| **A2503** | - A2503C leads to increased resistance to proline-rich antimicrobial peptide Onc112 (especially when combined with A2059C). - A2503G confers a small susceptibility to linezolid antibiotic in *M. smegmatis* (*E. coli* mutation unstudied) - A2503U confers resistance to valnemulin, chloramphenicol, florfenicol, tylosin, spiramycin, josamycin, and linezolid in *M. smegmatis* (*E. coli* mutation unstudied) | - Ganon, M.G., et al. NAR. 2016 44: 2439-2450. - Long, K.S., et al. Antimicrob Agents Chemother. 2010. 54(11): 4705-4713. - Li, B.B., et al. J. Antimicrob. Chemother. 2011. 66(9):1983-6. |
| **U2504** | - U2504A confer chloramphenicol resistance in mitochondrial rRNA (*E. coli* mutation unstudied). - U2504G growth rate increases to 5.4 h. Cross resistance to both chloramphenicol and linezolid in *M. smegmatis* (*E. coli* mutation unstudied). - U2504C confers linezolid resistance in *H. halobium* (*E. coli* mutation unstudied). | - Long. K.S., et al. Molecular Microbiology. 2009. 71(5): 1218-1217. |
| **G2505** | - G2505A confers resistance to oxazolidinone linezolid in Enterococcus faecalis. *E. coli* mutation studied in vitro shows 14% peptidyl transferase activity. Severe to lethal growth defects in vivo. - G2505U shows less than 5% peptidyl transferase activity in vitro. Severe to lethal growth defects in vivo. - G2505C shows 17% peptidyl transferase activity in vitro. Severe to lethal growth defects in vivo. When combined with A1067U, mutation is dominant lethal. | - Bourgeois-Nicolaos, N. et al. J. Infect. Dis. 2007. 195(10): 1480-8. - Saarma, U., and Remme, J. NAR. 1992. 20: 3147-3152. - Porse, B.T., et al. JMB. 1996. 264: 472-483. |
| **U2506** | - U2506A impacts ability to catalyze peptidyltransferase with Puromycin. Retains 5% peptidyl transferase activity in vitro. Lethal in vivo. - U2506G displays a cold-sensitive phenotype in which peptide bond formation is efficient at higher temperatures. Retains less than 5% peptidyl transferase activity in vitro. Lethal in vivo. - U2506C impacts ability to catalyze peptidyltransferase with Puromycin. Retains 20% peptidyl transferase activity in vitro. Lethal in vivo. | - Youngman, E.M., et al. Cell. 2004. 117(5): 589-599. - Porse, B.T., et al. JMB. 1996. 264: 472-483. |
| **C2507** | - C2507A remains unstudied - C2507U dominant lethal *in vivo*. - C2507G remains unstudied | - Spahn, C., et al. JBC. 1996. 271; 32849-32856. |
| **G2582** | - G2582A decreased total protein synthesis by approximately one-third, but not the RNA synthesis. Additionally, this mutation results in an increase in peptidyl-tRNA drop-off, thereby reducing translational processivity. Shows less than 5% peptidyl transferase activity in vitro. Lethal in vivo. - G2582U causes a significant increase in peptidyl-tRNA drop-off from ribosomes, thereby reducing translational processivity. Retains less than 5% peptidyl transferase activity in vitro. Lethal in vivo - G2582C retains <5% peptidyl transferase activity in vitro. Lethal in vivo. | - Maivali, U., et al. Mol. Biol. (Mosk). 2001. 35(4): 666-71. - Porse, B.T., et al. JMB. 1996. 264: 472-483. |
| **G2583** | - G2583A readily incorporates into 70S ribosomes and polysomes. When combined with A1067U, mutation is dominant lethal. Alone, mutation is results in very little activity in vitro (less than 5%), growth defects in vivo. - G2583U retains less than 5% peptidyl transferase activity in vitro. Lethal in vivo. - G2583C decreased total protein synthesis by approximately one-third, but not the RNA synthesis. Additionally, this mutation results in an increase in peptidyl-tRNA drop-off, thereby reducing translational processivity. Other studies show this mutation has <5% or no peptidyl transferase activity in vitro. Lethal in vivo. | - Saarma, U., et al. RNA. 1998. 4:189-194. - Saarma, U., and Remme, J. NAR. 1992. 20: 3147-3152. - Maivali, U., et al. Mol. Biol. (Mosk). 2001. 35(4): 666-71. - Porse, B.T., et al. JMB. 1996. 264: 472-483. |
| **U2584** | - U2584A retains 22% peptidyl transferase activity in vitro. Lethal in vivo. - U2584G dominant lethal in vivo. Retains 32% peptidyl transferase activity in vitro. - U2584C retains 21% peptidyl transferase activity in vitro. Some growth defect in vivo. | - Porse, B.T., and Garrett, R.A. JMB. 1995. 249:1-10. - Maivali, U., et al. Mol. Biol. (Mosk). 2001. 35(4): 666-71. |
| **U2585** | - U2585A lethal in vivo. Causes degradation of both large and small ribosomal subunits in E. coli. Retains 6% peptidyl transferase activity in vitro. - U2585G lethal *in vivo*. Peptidyltransferase rate constant is diminished in vitro by ~7-fold. Retains 36% peptidyl transferase activity in vitro. - U2585C lethal *in vivo*. Peptide release is compromised *in vitro*. Retains less than 5% peptidyl transferase activity in vitro. | - Paier, A., et al. Sci. Rep. 2015. 5:7712. - Youngman, E.M., et al. Cell. 2004. 117: 589-599. - Porse, B.T., et al. JMB. 1996. 264: 472-483. |
| **U2586** | - U2586A does not affect wild-type ErmCL peptide stalling. - U2586G does not affect wild-type ErmCL peptide stalling. - U2586C does not affect wild-type ErmCL peptide stalling. | - Arenz, S., et al. Molecular Cell. 2014. 56: 446-452. |
| **A2587** | - A2587C remains unstudied. - A2587G remains unstudied. - A2587U remains unstudied. |  |
| **G2588** | - G2588A alters the interaction of protein substrates and the antiprion compond 6-Aminophenanthridine (6AP) with domain V rRNA, and also decreases protein folding activity of the ribosome (PFAR) - G2588U remains unstudied. - G2588C remains unstudied. | - Pang, Y., et al. JBC. 2013. 288(26): 19081-19089. |
| **A2602** | - A2602C completely eliminates RF1-dependent peptidyl-tRNA hydrolysis. - A2602G has diminished peptide release activity. - A2602U has diminished peptide release activity. | - Polacek, N., et al. Molecular Cell. 2003. 11(1):103-112. |
| **C2606** | - C2606A remains unstudied. - C2606U remains unstudied. - C2606G remains unstudied. |  |
| **G2607** | - G2607A remains unstudied. - G2607U remains unstudied. - G2607C remains unstudied. |  |
| **G2608** | - G2608A does not confer resistance to oxazolidinone antibiotic. - G2608U confers resistance to oxazolidinone antibiotic. - G2608C confers strong resistance to oxazolidinone antibiotic. | - Xu, J., et al. Biochemical and Biophysical Research Communications. 2005. 328(2): 471-476. |
| **U2609** | - U2609A results in resistance to klebsazolicin (KLB). - U2609G results in resistance to klebsazolicin (KLB). - U2609C renders *E. coli* resistant to ketolides telithromycin and cethromycin | - Metelev, M., et al. Nature Chemical Biology. 2017. 13: 1129-1136. - Xiong, L., et al. Antimicrob Agents Chemother. 2005. 49(1): 281-288. |
| **C2610** | - C2610A remains unstudied. - C2610U in *S. pneumoniae* confers small impacts on the activities of macrolides and clindamycin, but is not categorized as resistance to these antimicrobials. Reduces Erythromycin-dependent ribosome stalling of ErmCL peptide in *E. coli.* - C2610G is associated with linezolid resistance in *E. faecalis*. Remains unstudied in *E. coli*. | - Canu, A., et al. Antimicrob. Agents. Chemother. 2002. 46(1): 125-131. - Vazquez-Laslop, N. et al. PNAS. 2011. 108: 10496-10501. - Boumghar-Bourtchai, L., et al. Antimicrob. Agents. Chemother. 2009. 53(9): 4007-4009. |
| **C2611** | - C2611A confers resistance to erythromycin and 14- and 15- membered macrolides in pneumococcal strains. - C2611U in *S. pneumoniae* confers small impacts on the activities of macrolides and clindamycin, but is not categorized as resistance to these antimicrobials. Remains unstudied in *E. coli*. - C2611G confers resistance to erythromycin and 14- and 15- membered macrolides in pneumococcal strains. | - Canu, A., et al. Antimicrob. Agents. Chemother. 2002. 46(1): 125-131. - Tait-Kamradt, A., et al. Antimicrob. Agents. Chemother. 2000. 44(8): 2118-2125. |

**Supplementary Table S2.** Bulk translation rates of wild-type and PTC-ring mutant 70S iSAT ribosomes. Bulk translation rates for iSAT ribosomes were determined from protein synthesis kinetics curves, for reactions after 2 h incubations, and normalized to wild-type.

| **Mutant** | **Average µM sfGFP** | **Bulk translation rate (µM protein/hr)** | **Std. Dev** |
| --- | --- | --- | --- |
| wt pT7rrnb | 1.00 | 1.03 | 0.03 |
| G2057U | 0.77 | 0.63 | 0.02 |
| G2057C | 0.57 | 0.52 | 0.01 |
| G2057A | 0.47 | 0.33 | 0.02 |
| A2058C | 0.89 | 0.69 | 0.03 |
| A2058U | 0.82 | 0.81 | 0.03 |
| A2058G | 0.57 | 0.51 | 0.02 |
| A2059G | 0.4 | 0.26 | 0.01 |
| A2059U | 0.22 | 0.33 | 0.02 |
| A2059C | 0.18 | 0.09 | 0.01 |
| A2060G | 0.27 | 0.17 | 0.01 |
| A2060U | 0 | 0 | 0.01 |
| A2060C | 0 | 0.02 | 0 |
| G2061A | 0.23 | 0.35 | 0.02 |
| G2061C | 0.06 | 0.02 | 0.01 |
| G2061U | 0 | 0.02 | 0 |
| A2062U | 0.94 | 1.02 | 0.02 |
| A2062C | 0.72 | 0.46 | 0.02 |
| A2062G | 0.63 | 0.43 | 0.02 |
| C2063U | 0 | 0 | 0 |
| C2063G | 0 | 0 | 0 |
| C2063A | 0 | 0 | 0 |
| G2447A | 0.44 | 0.2 | 0.01 |
| G2447C | 0.25 | 0.17 | 0.01 |
| G2447U | 0.01 | 0 | 0 |
| A2448C | 0.49 | 0.3 | 0.04 |
| A2448U | 0.48 | 0.21 | 0.02 |
| A2448G | 0.37 | 0.28 | 0.04 |
| U2449C | 0.64 | 0.52 | 0.02 |
| U2449A | 0.12 | 0.02 | 0 |
| U2449G | 0.02 | 0.01 | 0.01 |
| A2450G | 0.02 | 0 | 0 |
| A2450U | 0 | 0 | 0 |
| A2450C | 0 | 0 | 0 |
| A2451C | 0.36 | 0.15 | 0.01 |
| A2451U | 0.1 | 0.14 | 0.01 |
| A2451G | 0.02 | 0 | 0 |
| C2452A | 0.61 | 0.52 | 0.02 |
| C2452G | 0.03 | 0 | 0.01 |
| C2452U | 0.02 | 0.02 | 0.02 |
| A2453U | 0.57 | 0.34 | 0.03 |
| A2453C | 0.15 | 0.05 | 0.02 |
| A2453G | 0 | 0.01 | 0 |
| G2454A | 0.13 | 0.09 | 0.02 |
| G2454U | 0.04 | 0.01 | 0 |
| G2454C | 0 | 0 | 0 |
| G2455U | 0.27 | 0.16 | 0.01 |
| G2455A | 0.06 | 0.13 | 0.01 |
| G2455C | 0.02 | 0.02 | 0 |
| C2456U | 0.66 | 0.43 | 0.1 |
| C2456G | 0.09 | 0.06 | 0 |
| C2456A | 0.08 | 0.06 | 0 |
| C2496U | 0.9 | 0.74 | 0.02 |
| C2496G | 0.38 | 0.31 | 0.02 |
| C2496A | 0.16 | 0.11 | 0.01 |
| A2497U | 0.67 | 0.48 | 0.01 |
| A2497G | 0.26 | 0.12 | 0.01 |
| A2497C | 0.1 | 0.13 | 0.01 |
| C2498U | 0.06 | 0 | 0 |
| C2498A | 0 | 0 | 0.01 |
| C2498G | 0 | 0 | 0.02 |
| C2499G | 0.04 | 0.03 | 0.01 |
| C2499U | 0.01 | 0 | 0 |
| C2499A | 0.01 | 0.03 | 0 |
| U2500A | 0.36 | 0.3 | 0.03 |
| U2500G | 0 | 0.01 | 0 |
| U2500C | 0 | 0.06 | 0.02 |
| C2501U | 0.03 | 0.02 | 0.01 |
| C2501G | 0 | 0 | 0 |
| C2501A | 0 | 0 | 0 |
| G2502U | 0.29 | 0.19 | 0.01 |
| G2502C | 0.04 | 0.02 | 0.01 |
| G2502A | 0.02 | 0.01 | 0 |
| A2503U | 0.79 | 0.64 | 0.02 |
| A2503G | 0.28 | 0.34 | 0.02 |
| A2503C | 0.01 | 0.03 | 0.02 |
| U2504G | 0.6 | 0.4 | 0.01 |
| U2504A | 0.35 | 0.19 | 0 |
| U2504C | 0.21 | 0.16 | 0.01 |
| G2505U | 0.98 | 0.85 | 0.02 |
| G2505A | 0.1 | 0.05 | 0 |
| G2505C | 0.01 | 0.05 | 0.1 |
| U2506G | 0.03 | 0.02 | 0.02 |
| U2506C | 0.02 | 0.01 | 0.01 |
| U2506A | 0.02 | 0.02 | 0.01 |
| C2507U | 0.15 | 0.05 | 0.01 |
| C2507A | 0 | 0 | 0 |
| C2507G | 0 | 0.01 | 0 |
| G2582U | 0.34 | 0.37 | 0.03 |
| G2582A | 0.02 | 0 | 0 |
| G2582C | 0 | 0 | 0 |
| G2583A | 0.82 | 0.53 | 0.02 |
| G2583U | 0.07 | 0.03 | 0 |
| G2583C | 0.01 | 0 | 0 |
| U2584A | 0.66 | 0.44 | 0.02 |
| U2584C | 0.56 | 0.38 | 0.02 |
| U2584G | 0.25 | 0.11 | 0.02 |
| U2585G | 0.21 | 0.2 | 0.01 |
| U2585A | 0.02 | 0.02 | 0.03 |
| U2585C | 0.02 | 0.01 | 0.01 |
| U2586G | 0.57 | 0.52 | 0.01 |
| U2586A | 0.47 | 0.36 | 0 |
| U2586C | 0.44 | 0.31 | 0.04 |
| A2587U | 0.83 | 0.8 | 0.04 |
| A2587G | 0.2 | 0.12 | 0.01 |
| A2587C | 0.09 | 0.04 | 0.01 |
| G2588U | 0.56 | 0.38 | 0.01 |
| G2588C | 0.47 | 0.49 | 0.02 |
| G2588A | 0.35 | 0.28 | 0.01 |
| A2602U | 0.03 | 0.02 | 0 |
| A2602G | 0.02 | 0.01 | 0.01 |
| A2602C | 0.02 | 0.01 | 0 |
| C2606U | 0.42 | 0.3 | 0.01 |
| C2606A | 0.2 | 0.15 | 0.01 |
| C2606G | 0.15 | 0.05 | 0.02 |
| G2607C | 0.5 | 0.45 | 0.02 |
| G2607U | 0.47 | 0.19 | 0.01 |
| G2607A | 0.2 | 0.15 | 0.01 |
| G2608A | 0.9 | 0.58 | 0.02 |
| G2608U | 0.78 | 0.67 | 0.02 |
| G2608C | 0.77 | 0.57 | 0.03 |
| U2609A | 0.8 | 0.55 | 0.01 |
| U2609C | 0.74 | 0.47 | 0.02 |
| U2609G | 0.68 | 0.39 | 0.03 |
| C2610G | 0.41 | 0.49 | 0.02 |
| C2610U | 0.33 | 0.25 | 0.02 |
| C2610A | 0.04 | 0 | 0 |
| C2611U | 0.55 | 0.42 | 0.02 |
| C2611A | 0.11 | 0.03 | 0.01 |
| C2611G | 0.03 | 0 | 0 |

**Supplementary Table S3. Equations and examples scores for relative activity calculations, and overall mutational flexibility scores.** Relative activity was calculated to compare performance of each mutant by normalizing wild-type protein synthesis yields to one and mutant yields to the normalized wild-type yields. An overall mutational flexibility score was then determined for each nucleotide position by adding the relative activities of every possible point mutation. The highest mutational flexibility score of three indicates that all three nucleotide changes possess wild-type activity, while the lowest mutational flexibility score of zero indicates that all three nucleotide changes preclude any protein synthesis.

| **Value/Score** | **Equation used** | **Example calculations** | |
| --- | --- | --- | --- |
| Relative activity | $R=\frac{1}{WT}*(Mutant activity)$ | **Protein synthesis yields**  **WT:** 9.6 µM  **A2062U:** 9.0 µM  **A2062C:** 6.9 µM  **A2062G:** 6.0 µM | **Relative activity**  **WT:** 1  **A2062U:** 0.94  **A2062C:** 0.72  **A2062G:** 0.63 |
| Mutational flexibility | $R1+R2+R3$ | **Relative activity**  **A2062U:** 0.94  **A2062C:** 0.72  **A2062G:** 0.63 | **Mutational flexibility**  **A2062:** 2.3 |

**Supplementary Table S4. Sucrose gradient fractionation profiles of rRNA PTC mutants.** Representative nucleotide mutations were chosen for sucrose gradient fractionation based on their activity. iSAT reactions were separated through sucrose gradients, and fractions were collected. The relative average abundance of rRNA in each fraction was quantified by calculating the area under each curve.

| Ribosomes | 30S | 50S | 70S | Disomes | Trisomes |
| --- | --- | --- | --- | --- | --- |
| WT | 34% | 25% | 14% | 15% | 12% |
| A2062U | 36% | 30% | 12% | 12% | 8% |
| U2585G | 56% | 26% | 9% | 9% | 0% |
| A2451U | 50% | 41% | 7% | 2% | 0% |
| G2455A | 46% | 48% | 4% | 2% | 0% |

**Supplementary Table S5.** PTC-ring nucleotide distances calculated in PyMol. PTC nucleotide distances were calculated from either the A-site or P-site tRNAs. Using the *E. coli* ribosome structure (PDB- 4YBB), we measured the distance between A76 of the A-site and P-site tRNAs and within one angstrom of the geometric center of each PTC nucleotide.

| **A site (A76 of A-site tRNA + AA)** | | **P site (A76 of P-site tRNA + AA)** | |
| --- | --- | --- | --- |
| Distance | Nucleotides | Distance | Nucleotides |
| 2 Å or less | 2583 | 3 Å or less | 2062, 2585 |
| 3 Å or less | 2506, 2451, 2585 | 4 Å or less | 2061, 2063, 2450, 2451 |
| 4 Å or less | 2584, 2452 | 5 Å or less | 2586, 2602 |
| 5 Å or less | 2061, 2505, 2507 | 6 Å or less | 2584 |
| 6 Å or less | 2063, 2504, 2582 | 7 Å or less | 2452 |
| 7 Å or less | 2447, 2450 | 8 Å or less | 2503, 2505, 2506 |
| 8 Å or less | 2501, 2503, 2453, 2602 | 9 Å or less | 2447, 2501, 2504, 2583, |
| 9 Å or less | 2500 | 10 Å or less | 2497, 2587, 2608 |
| 10 Å or less | 2062 | 11 Å or less | 2059, 2449 |
| 11 Å or less | 2586, 2610 | 12 Å or less | 2453, 2507 |
| 12 Å or less | 2059, 2454, 2499, 2608 | 13 Å or less | 2496, 2500, 2609, 2610 |
| 13 Å or less | 2058, 2449, 2497 | 14 Å or less | 2058, 2502, 2588, 2607 |
| 14 Å or less | 2502, 2496, 2607, 2611 | 15 Å or less | 2060, 2499, 2582, 2606, 2611 |
| 15 Å or less | 2057, 2455, 2498, 2587, 2606 | 16 Å or less | 2057, 2448, 2454, 2455, 2498 |
| 16 Å or less | 2060, 2588 |  |  |
| 17 Å or less | 2448, 2456, 2609 |  |  |

**Supplementary Table S6.** A- and P-loop nucleotide distances calculated in PyMol. Distances were calculated from either the A-site or P-site tRNAs. Using the *E. coli* ribosome structure (PDB- 4YBB), we measured the distance between A76 of the A-site and P-site tRNAs and within one angstrom of the geometric center of each PTC nucleotide.

| **A site (A76 of A-site tRNA + AA)** | | **P site (A76 of P-site tRNA + AA)** | |
| --- | --- | --- | --- |
| Distance | Nucleotides | Distance | Nucleotides |
| 4 Å or less | 2553 | 4 Å or less | 2251 |
| 7 Å or less | 2554 | 7 Å or less | 2252 |
| 10 Å or less | 2552, 2555 | 10 Å or less | 2253 |
| 12 Å or less | 2251 | 13 Å or less | 2250, 2254 |
| 14 Å or less | 2556 | 15 Å or less | 2553 |
| 15 Å or less | 2252 | 17 Å or less | 2554 |
| 17 Å or less | 2253, 2557 | 19 Å or less | 2555 |
| 18 Å or less | 2551 | 21 Å or less | 2552 |
| 19 Å or less | 2250 | 23 Å or less | 2556 |
| 20 Å or less | 2550, 2558 | 26 Å or less | 2557 |
| 21 Å or less | 2254 | 28 Å or less | 2551 |
| 23 Å or less | 2549 | 31 Å or less | 2558 |
| 24 Å or less | 2559 | 32 Å or less | 2550 |
| 25 Å or less | 2548 | 33 Å or less | 2549 |
| 26 Å or less | 2560 | 34 Å or less | 2559, 2548 |
|  |  | 36 Å or less | 2560 |
